# Metal Lability and Mass Transfer Response to Direct-Planting Phytostabilization of Pyritic Mine Tailings

**DOI:** 10.3390/min12060757

**Published:** 2022-06-15

**Authors:** Corin M. Hammond, Robert A. Root, Raina M. Maier, Jon Chorover

**Affiliations:** Department of Environmental Science, University of Arizona, Tucson, AZ 85721, USA;

**Keywords:** metal lability, lead, zinc, mine tailings, phytoremediation, phytostabilization

## Abstract

Understanding the temporal effects of organic matter input and water influx on metal lability and translocation is critical to evaluate the success of the phytostabilization of metalliferous mine tailings. Trends of metal lability, e.g., V, Cr, Mn, Co, Ni, Cu, Zn, and Pb, were investigated for three years following a direct-planting phytostabilization trial at a Superfund mine tailings site in semi-arid central Arizona, USA. Unamended tailings were characterized by high concentrations (mmol kg^−1^) of Fe (2100), S (3100), As (41), Zn (39), and Pb (11), where As and Pb greatly exceeded non-residential soil remediation levels established by Arizona. Phytostabilization treatments included a no-compost control, 100 g kg^−1^ compost with seed, and 200 g kg^−1^ compost with and without seed to the top 20 cm of the tailings profile. All plots received supplemental irrigation, effectively doubling the mean annual precipitation. Tailings cores up to 90 cm were Collected at the time of planting and every summer for 3 years. The cores were sub-sectioned at 20 cm increments and analyzed via total digestion and an operationally defined sequential extraction for elemental analysis and the calculation of a mass transfer coefficient normalized to Ti as an assigned immobile element. The results indicate that Pb was recalcitrant and relatively immobile in the tailings environment for both the uncomposted control and composted treatments with a maximum variation in the total concentration of 9–14 mmol kg^−1^ among all samples. Metal lability and translocation above the redox boundary (*ca*. 30 cm depth) was governed by acid generation, where surficial pH was measured as low as 2.7 ± 0.1 in year three and strongly correlated with the increased lability of Mn, Co, Ni, Cu, and Zn. There was no significant pH effect on the lability of V, Cr, or Pb. Translocation to depths was greatest for Mn and Co; however, Zn, Ni, Cr, and Cu were also mobilized. The addition of organic matter enhanced the mobilization of Cr from the near surface to 40–60 cm depth (pH > 6) over the three-year phytostabilization study compared to the control. The increased enrichment of some metals at 60–90 cm indicates that the long-term monitoring of elemental translocation is necessary to assess the efficacy of phytostabilization to contain subsurface metal contaminants and thereby protect the surrounding community from exposure.

## 1. Introduction

Abandoned, metalliferous, sulfide mine tailings pose a major human health and environmental hazard due to anthropogenic disturbance and the associated introduction of contaminant-bearing minerals into the environment [1–3]. Legacy tailings exhibit nutrient deficiency (C, N, and P), acidic pH, elevated salt levels, and high concentrations of hazardous metals and metalloids such as lead, zinc, copper, mercury, cadmium, and arsenic [4], Together, these characteristics make tailings an inhospitable environment for plant and heterotrophic microbial growth [5–8], which keeps them devoid of vegetation establishment for decades to centuries following mining cessation, particularly in (semi-) arid regions. As a result, tailings are highly susceptible to fugitive dust emissions and rainwater runoff erosion, leading to the exposure of wildlife and humans to toxic elements [5,7,9–11]. The ever-increasing demand for mining commodities requires corresponding research into improved long-term and affordable management strategies for mine waste containment to avoid the degradation of the proximal environment; the development of such techniques is an essential component of environmentally sustainable mining strategies.

Phytostabilization is such a strategy, offering long-term, vegetation-driven containment to sequester and immobilize contaminants in the subsurface and rhizosphere under an established vegetation cap [3,12–14]. The growth of vegetation cover is key to providing the long-term physical stabilization of the tailings. The cover decreases wind shear at the surface and provides stabilization of the tailings, reducing fugitive emissions of particulate matter. Ferric (sulf)hydroxide crusts, which have high affinity for metal(loid) sorption, can form on root surfaces in mine tailings and sequester labile metal(loids) [12]. Additionally, roots provide a supportive scaffold that reduces wind and water erosion, and transpiration from vegetation diminishes contaminant leaching [4], Many areas experiencing heavy mining activity are located in (semi-) arid regions where water throughflux is limited, and therefore, so is the production of acid mine drainage (AMD). Dry conditions lead to the persistence of secondary weathering minerals, metal contaminants, and acidity in tailings media, preventing natural plant establishment [3,15,16]. Secondary minerals in mine tailings play important roles in contaminant retention and stabilization [17], For example, sulfates and (oxyhydr)oxides have been shown to attenuate solubilized metals in acid mine drainage [18–22].

Successful phytostabilization causes persistent organic root exudates and leaf litter to serve as a carbon source to the tailing ecosystem, with positive feedback on developing soil characteristics. The aim of phytostabilization is to achieve a self-sustaining ecosystem that eschews additional (and costly) inputs. The key to the success of phytostabilization is the negligible accumulation of toxic elements into the food-web in the above-ground biomass to reduce contaminant exposure to local wildlife [8,13,14], Given the low pH, low nutrient capacity, and poor soil structure of sulfide-ore-derived mine tailings, amendments such as irrigation, biosolids, fertilizers, lime, and chemical adsorbents are frequently employed to enhance plant establishment and growth [14,23–27], Despite an interest in phytostabilization as a remediation approach for mine tailings, the availability of published, long-term (≥2 years) field studies that assess the impacts of phytostabilization of metals contained in mine waste in (semi-) arid climates is lacking [28–32], Among long-term field-scale phytostabilization studies, this is the first to assess metal lability and translocation across both temporal and spatial variances. Elements of interest for this study include those in high concentrations and trace levels. The Iron King Mine Humboldt Smelter site (IKMHSS) tailings contain high zinc and manganese levels, which are both essential micronutrients but are toxic at high activities, and high lead levels, a toxic metal with no safe concentration level. Nickel, copper, chromium, vanadium, and cobalt, which are also micronutrients with toxic potential, are present in the tailings, but in much lower concentrations (<4 mmol kg^−1^). The comparative analysis of enriched and trace element lability and translocation provided a comprehensive dataset to assess geochemical processes taking place in the subsurface. The principal aims of this investigation were to (i) evaluate changes in transition metal (V-Zn) and Pb lability and translocation at the field-scale in response to the phytostabilization of sulfidic tailings from the surface to 90 cm deep and (ii) assess phytostabilization as an effective long-term stabilization strategy for these metals in tailings in a (semi-) arid climate over three years of tailings remediation.

The IKMHSS acid sulfide tailings [33], listed due to high arsenic and lead concentrations, are dominated by iron and sulfur (13 wt% and 12 wt% in the top 0.5 m, respectively) [17,34]. The oxidative dissolution of initially deposited pyrite (FeS_2_) releases iron, sulfate, acid, and toxic elements, which drives the precipitation of secondary ferric minerals such as jarosite (KFe_3_^+3^(OH)_6_(SO_4_)_2_), ferrihydrite (Fe_2_^3+^O_3_·0.5(H_2_O), and schwertmannite (Fe_16_^3+^O_16_(OH)_12_(SO_4_)_2_). The wind-driven erosion of the IKMHSS surface tailings is known to transport toxic metal(loid)-containing particulates to the surrounding community [5,8,10,35,36], Toxic metal(loid) lability and transport are of particular concern at this site due to arsenic concentrations being measured above the allowed state limit for residential soils (10 mg kg^−1^) [37], The field-scale (appx. 1 ha) phytostabilization of the IKMHSS tailings, initiated in 2010, established the long-term feasibility and efficacy of the compost-assisted, direct planting of a vegetation cap to reduce off-site dust emissions and sequester toxic metalloids in place [4,38], Previous studies at this site showed that phytoremediation was especially effective in reducing emissions of fine particulate matter (e.g., PM_2.5_), which have the greatest health risks and the highest potential for long-distance transport [38], The IKMHSS tailings remedial-strategy investigation of the subsurface fate and mobility of hazardous metals is a case study of the fate and mobility of metal contaminants in acidic sulfide mine waste in (semi-) arid regions worldwide.

The tailings at IKMSS were sourced from regional (mostly Pb, Zn) mines (<100 km) with the mineralogy dominated by pyrite (FeS_2_), ankerite (Ca[Fe, Mg](CO_3_)_2_), and quartz (SiO_2_). Valuable ores in the area included chalcopyrite (CuFeS_2_), galena (PbS), sphalerite (ZnS), and tennantite ([Cu, Fe]_12_As_4_S_13_), as sources of zinc and lead, while gold and silver, occluded in pyrite and tennantite, respectively, were mined as secondary commodities [39]. The dominant non-sulfide “gangue” minerals (those having no commercial value) were ankerite, quartz, pyrite (up to 75% of the original vein), and arsenopyrite (FeAsS) [39]. The tailings have been weathering in place, unamended for half a century following the mine’s closure. A redox gradient with a boundary at ca. 30 cm depth with a transition zone from 25 to 54 cm depth was identified and associated with distinct depth-dependent geochemical and morphological changes. Surficial tailings were characterized by (oxyhydr)oxide and sulfate minerals (*ex*. jarosite, ferrihydrite, schwertmannite, zincosite [ZnSO_4_], and gypsum [CaSO_4_·2H_2_O]), and metal(loid)s adsorbed to jarosite and ferrihydrite. Tailings deeper than 55 cm were characterized by carbonates and sulfides [17,34].

We hypothesized that the addition of labile carbon and a vegetative cap would help to buffer a more neutral pH, relative to unamended tailings, and decrease metal solubility. It was established that near-surface ferric (sulf)oxyhydroxide minerals with high adsorption capacity for metals sequestered arsenic and could also decrease metal mobility [4]. Therefore, it was expected that compost-assisted vegetation plots would show greater metal retention in the rhizosphere compared to unamended control plots due to plant-promoted pH buffering, sorption on existing ferric hydroxide surface sites, and organo-metallic complexation.

## 2. Materials and Methods

### 2.1. Field Site Experimental Setup

The field-scale experiment investigating the direct planting of phytostabilization in IKMHSS mine tailings at the Superfund Site in Dewey-Humboldt, Arizona, USA, was initiated in 2010. The geochemical properties, field site description, and phytostabilization experiment design have been previously described [4,6,17,34,40]. In brief, a three-year field-scale phytoremediation study was carried out using native halotolerant plant species and six treatments of 0% compost without seed (control), 10% (100 g kg^−1^ and 228 t ha^−1^) compost with seed, 15% (150 g kg^−1^ and 342 t ha^−1^) compost without seed, 15% compost with seed, 20% (200 g kg^−1^ and 456 t ha^−1^) compost without seed, and 20% compost with seed, where compost addition was per unit mass of the top 20 cm of mine tailings. Treatments were randomly distributed and replicated in quadruplicate in a total of twenty-four 9.6 m × 15 m field plots (Figure S1). Each was bermed to ca. 50 cm to minimize cross contamination. Control plots were placed at the corners of the study area to eschew impacts from amendments. Prior to direct planting, tailings received a one-time addition of dairy manure-green waste compost from Arizona Dairy Compost LLC (Anthem, AZ, USA). The compost was weighed and tilled into each plot to a depth of ca. 20 cm. Straw was scattered at a rate of 61 ha^−1^ and crimped 10 cm into the tailings to decrease compaction by irrigation, seed predation by birds, dispersion by wind, and water evaporation. Native halotolerant plant species (seed source: Desert Nursery, Phoenix, AZ, USA) were selected based on high biomass production and low above-ground metal(loid) bioaccumulation [40]. The seeding rates were: *Festuca arizonica* (56 kg ha^−1^), *Buchloe dactyloides* (90 kg ha^−1^), *Acacia greggii* (1 kg ha^−1^), *Prosopis juliflora* (0.15 kg ha^−1^), *Cercocarpus montanus* (11 kg ha^−1^), and *Atriplex lentiformas* (56 kg ha^−1^). The 20% compost seeded plots received seed from all six species, while the 10% compost plots only received *Buchloe dactyloides* and *Prosopis juliflora*, which were determined by preliminary greenhouse studies to survive successfully at suboptimal compost application [40]. Seeds and straw were applied at night to avoid losses due to high daytime winds. The field site was irrigated every 7 to 10 days for three years with about 0.5 to 1 cm of water (322–387 mm annually), except during periods of appreciable rainfall or freezing temperatures [6].

### 2.2. Sample Collection and Storage

Tailing cores (2.54 cm diameter, up to 91.4 cm length) were collected at time 0 and annually in triplicate from every plot during the summer between late May and early July. Cores were transported on dry ice and stored at −15 °C until they were processed (Figure S2). Cores were sectioned at 20 cm increments starting at the surface (surface–20 cm, 20–40 cm, 40–60 cm, and 60 cm-bottom) in an anoxic glove bag (95% N_2_ and 5% H_2_). Recovery in some cores varied as a function of compaction in the deepest segment (60–91.4 cm). Each subsection was homogenized, freeze-dried, sieved to 2 mm, and stored at −15 °C for geochemical analyses.

### 2.3. Chemical Characterization

The tailings were analyzed for pH and EC from the supernatant of a 1:1 suspension of tailings: lab-pure water (Milli-Q, 18.2 MΩ-cm). Samples were mixed for 1 h in 40 mL polypropylene centrifuge tubes on a lateral shaker and then centrifuged at 42,000 RCF (18,000 rpm) for 5 min to isolate the supernatant according to EPA method 9045D. Metal lability and operationally defined solid-phase speciation were determined using a 3-step specific sequential extraction (SSE) method modified from Dold and Keon et al. [41,42], Samples were sequentially extracted at a 1:100 solid to solution mass ratio with (1) ultrapure water (DI, 18.2 MΩ-cm, 25 °C, unbuffered, 1 h) targeting soluble salts; (2) 1 M monosodium phosphate (NaH_2_PO_4_, 25 °C, pH 5.0, 24 h) targeting exchangeable and adsorbed ions; and (3) 0.2 M acid ammonium oxalate (Tamm’s Reagent, 25 °C, pH 3.0, 2 h, dark in Al foil), targeting poorly crystalline Al, Mn, and Fe (hydr)oxides via ligand-promoted dissolution (Table 1). Extractants were added to tailing subsamples in a stepwise fashion, with each step followed by centrifugation and the aspiration of the supernatant. Samples were washed using a reciprocal shaker with ultrapure water (18.2 MΩ-cm) after steps 2 and 3 for 30 min, and the wash was combined with the extract for analysis. Extractable pools are reported as the percent removed relative to the total concentration. The elemental analysis of sequential extractions, microwave-assisted aqua regia digestions, and microwave-assisted HF/HCI/HNO_3_ digestions were measured using inductively coupled plasma-mass spectrometry (ICP-MS, Perkin Elmer, Elan DRC-II) following extraction/digestion and appropriate dilution (max TDS 2500 mg kg^−1^) with 1% HNO_3_ (OmniTrace^®^) in the Arizona Laboratory for Emerging Contaminants (ALEC) at the University of Arizona. Extractions and digestions were performed using freshly prepared solutions (<24 h). All samples were run in field-plot quadruplicates with averages and standard deviations reported; field-scale variation greatly exceeded analytical variation, and field replicates were used for error analysis.

The unextracted residual concentration of each element was calculated using the mass balance from the total elemental analysis determined with microwave-assisted aqua regia digestion according to EPA Method 3051A. The digestion temperature in sealed microwave Teflon^®^ vessels was ramped for 5.5 min to 175 °C and held at a constant for 45 min prior to cooling. Separate HF/HCI/HNO_3_ microwave-assisted digestion was performed by applying a 1.0 g: 60.0 mL tailings: acid solution ratio (acid: 16.7% HF AriStar^®^ Ultra., 41.7% HC1 Aristar^®^ Plus, 41.7% HNO_3_ Aristar^®^ Plus). The digestion temperature was ramped for 15 min to 180 °C and held at a constant for 30 min prior to cooling. Extractions and digestions were centrifuged at 42,000 RCF (18,000 rpm) for 25 min followed by filtration with 0.2 μm GHP membranes, except in step 1 of SSE (DI H_2_O), in which they were centrifuged at 37,000 RCF (17,000 rpm) for 3 min to sediment the >0.125 μm size range, and HNO_3_/HCI/HF digestion, where vessels were settled for 24 h prior to careful aspiration to avoid spills. Montana Soil 2711a (NIST reference material) and IKMHSS tailings analyzed by an outside commercial laboratory (Activation Labs, Ontario, Canada) were used for comparative quality assurance [34]. The concentrations were within ±10% of the certified values for most elements. The percentage of deviation from certified values for Mn and Ti were observed to be at 13% and 17%, respectively.

To quantify metal translocation during the phytostabilization experiment, an enrichment and depletion index was calculated for depth profiles with a mass balance approach [43]. A sample from 180 cm was operationally defined as the parent material, and Ti was defined as the immobile element. The mass transfer coefficient (τ_j,W_) indicates the depletion (−τ)or enrichment (+τ) of element *j* relative to Ti at measured depths (Equation (1)).

(1) $$\tau_{Ti,j}=\left[\frac{C_{j,w}}{C_{j,p}}\times \frac{C_{Ti,p}}{C_{Ti,w}}\right]-1$$

where *C*_*j,w*_ and *C*_*j,p*_ are the concentrations of element *j* in the weathered tailings profile and the parent material, respectively, and *CT_i,w_* and *CT_i,p_* are the concentrations of Ti in the weathered zone and the parent material, respectively. Positive *τ* values reflect a fractional normalized gain of element *j* compared to the parent material, negative τ reflects a relative loss, and *τ*_*j,w*_ = 0 indicates no enrichment/depletion for element *j* in the weathering zone.

## 3. Results

### 3.1. Phytostablization Effect on Acidification and Buffering

**Figure 1.**
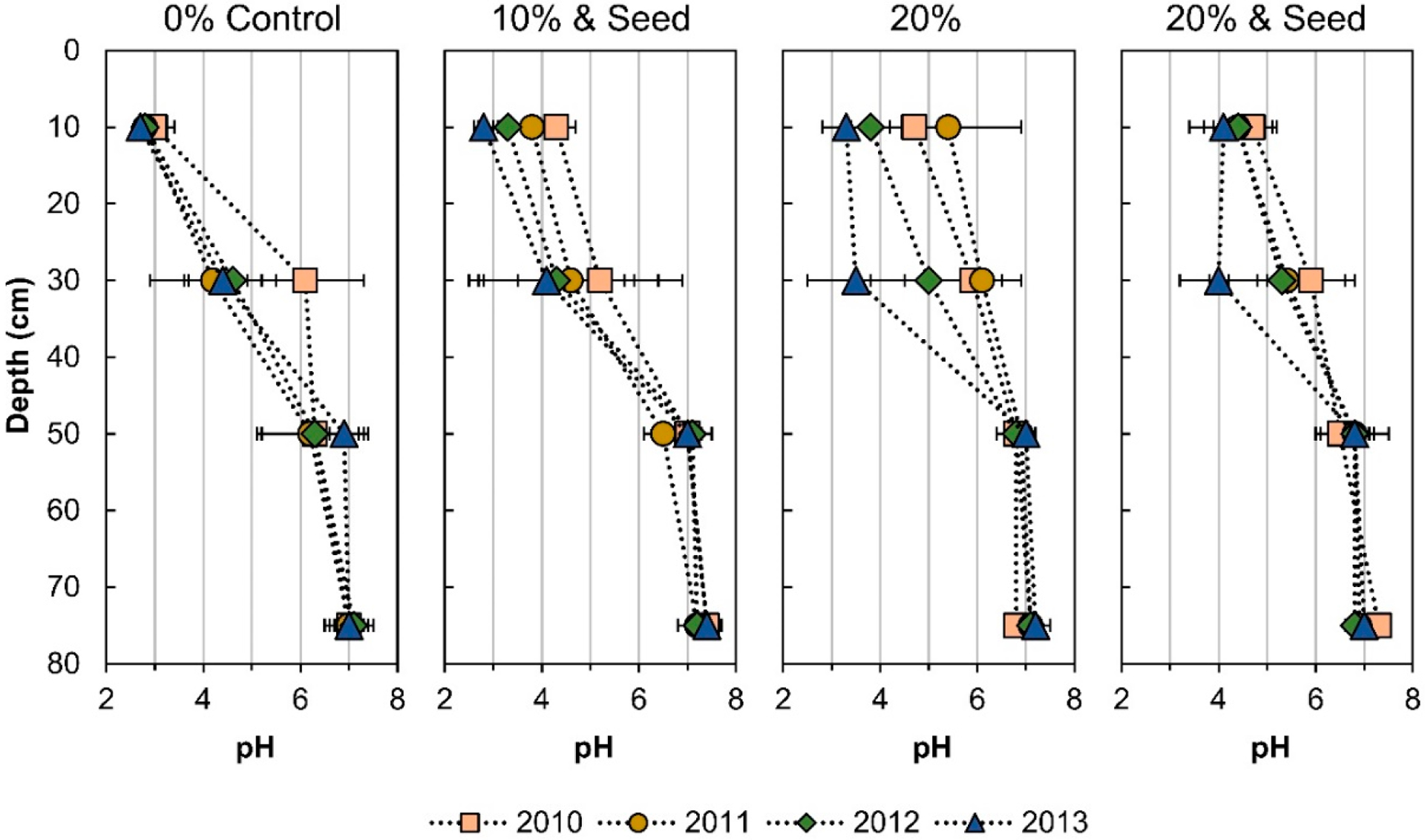
Tailings pH. Error bars are the standard deviation for *n* = 4 field replicates. Depth markers are the middle of the homogenized sectioned core, reported as cm below the surface.

For the three years of the study, seeded plants in compost-amended treatments survived. The plots with compost also showed growth in non-research plants, imported into the site by windblown or animal-promoted seed dispersal. The establishment of plants demonstrated that a direct benefit of phytoremediation was a substantial decrease in dust and the transport of windblown contaminants from the mine tailings [38]. The initiation of phytostabilization with compost amendments increased the pH of the near-surface tailings. However, each subsequent year, the surface tailings’ (0–20 cm) pH decreased (Figures 1 and S3). For the 20% compost treatment, surface tailing pH was stable or increased one year after planting, followed by progressive acidification. The 20% compost and seed treatment showed buffering against acidification (pH > 4) at the surface for 3 y and at 20–40 cm for 2 y. Acidification was observed at 20–40 cm for all treatments by 3 y while pH changes below 40 cm were not significant (Figure 1).

### 3.2. Metal Lability

**Figure 2.**
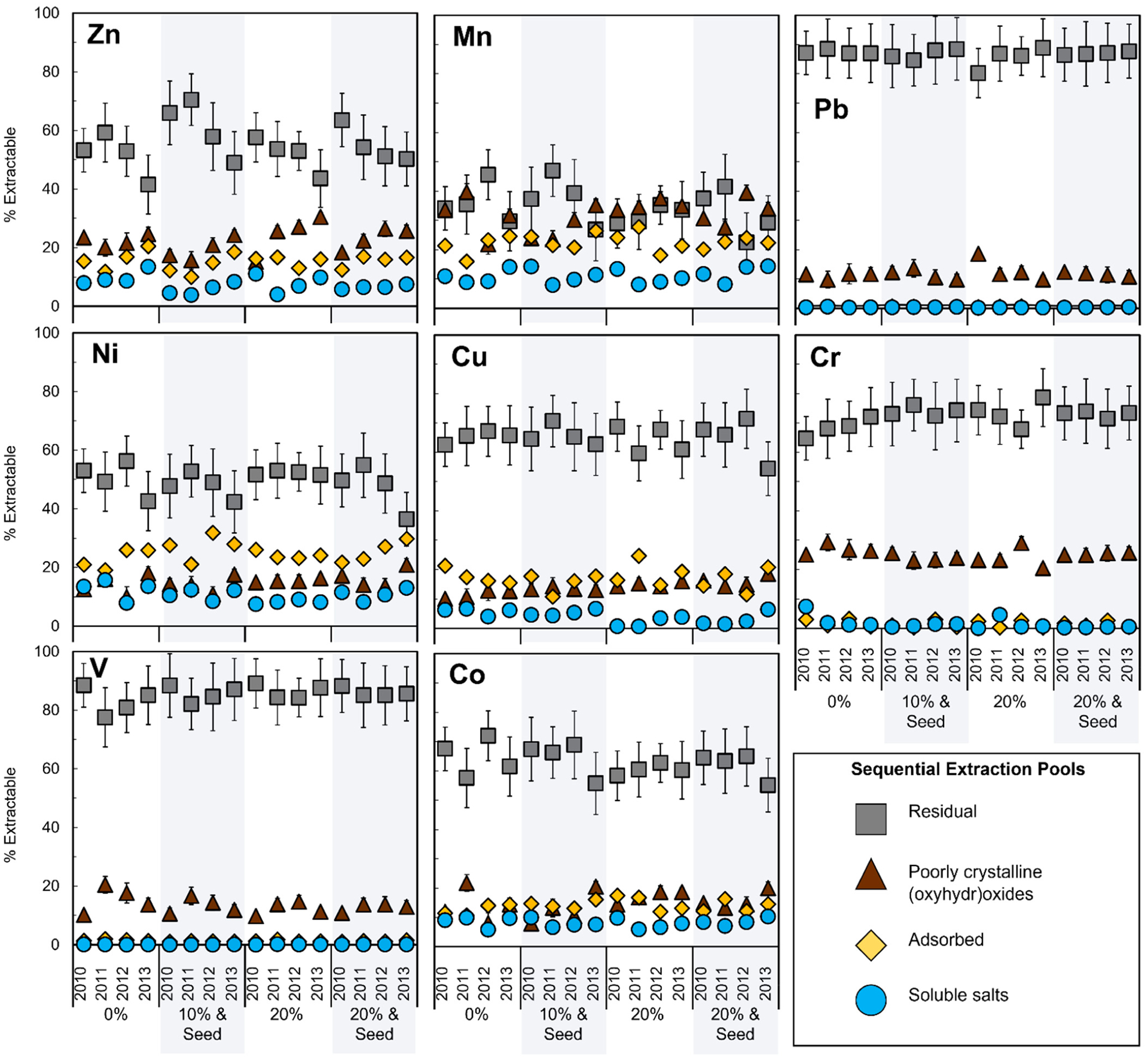
Depth-weighted average for the sequential extraction of metals. Extractable fractions of each element in operationally defined “water-soluble” (blue circle), “adsorbed” (yellow diamond), “poorly crystalline (oxyhydr)oxide” (brown triangle), and “residual” (gray square). Lability is reported as a percent of the total concentration for a full core length (0–90 cm) to account for the non-equivalent depth intervals (0–20, 20–40, 40–60, and 60–90 cm). Error bars denote standard deviation among quadruplicate field replicate cores (*n* = 4). Shaded regions indicate seeded treatments. Full sequential extraction and total aqua regia digestion datasets are provided in the Supplemental Information (Figures S4–S11 and Tables S1–S12).

Specific sequential extractions revealed element-specific variation in operationally defined pools of lability including the effects of time (3y), remediation (phytostabilization) (Figure 2), and depth (Figures S4–S11). Divalent metals Ni, Cu, and Zn had large labile fractions, defined here as SSE steps 1 + 2, the water-soluble plus adsorbed portions. Metals V, Cr, and Pb were mostly released in the recalcitrant pool, defined as SSE step 3 plus the residual pool, calculated using the mass balance from microwave digestion with aqua regia. Less than 3% of the metals were extracted in steps 1 + 2 and >50% remained after step 3 for all treatments and years (Figure 2).

#### 3.2.1. Depth Effect

Decreased lability with increased depth was observed for most metals in the control plots. Most commonly, increased depth was associated with a decreased water-soluble pool, e.g., Mn, Co, Ni, Cu, and Zn (Figures S6–S10). The magnitude of the water-soluble pool generally decreased with depth, and the adsorbed pool generally increased as the water-soluble pool decreased. However, as the Mn and Ni adsorbed pools increased with depth, the Cu adsorbed pool decreased. Increased depth was associated with a decrease in the poorly crystalline (oxyhydr)oxide-associated pool for V and a decreased residual pool for Mn (Figures S4 and S6). There was no significant depth-associated trend in the lability pools for Cr or Pb (Figures S5 and S11).

#### 3.2.2. Organic Matter Effect

Organic matter amendments had smaller effects on metal lability than depth. Compost addition did correlate with decreased lability (water-soluble and adsorbed fractions) for Mn and Zn, while Cu showed a decrease in lability with respect to the water-soluble fraction but displayed an increase in the adsorbed pool (Figures S6, S9 and S10). Compost amendment generally enhanced the poorly crystalline (oxyhydr)oxide pool, with the exceptions of Pb, which showed no significant effect from compost, and V, which showed a decrease in the poorly crystalline pool (Figures S4–S11).

#### 3.2.3. Temporal Effect

A decrease in the total Mn, Co, Ni, Cu, and Zn at 0–20 cm and 20–40 cm was observed over 3 y, consistent with the metals that showed a decrease in the water-soluble fraction with depth (Figures S6–S10). An increase in Mn at 40–60 cm in the 20% with seed treatment was observed with a concurrent near-surface decrease. The effect of weathering time on Ni generally showed translocation to greater depths. The elements that generally demonstrated recalcitrance or small effects with respect to depth and OM treatment, V, Cr, and Pb, also exhibited negligible temporal variation in lability.

### 3.3. Phytostabilization Effect on Mass Transfer

The mass transfer of metals was calculated with respect to immobile Ti in the unweathered parent material (Equation (1)). In the deepest core section (60–90 cm), an increase in metal concentration over the course of phytostabilization was observed for Mn, Co, Ni, Cu, and Cr (Figure S12). The temporal increase in metals over 3 y at the bottom of the collected cores was statistically significant despite the high field-scale variability in Mn and Co concentration, suggesting a downward translocation of Mn and Co from the irrigated surface due to metal lability in surficial tailings.

The total metal concentrations between the control and OM treatments at time 0 showed no significant difference (Figure S12). Whereas initial concentrations in the surface tailings (0–20 cm) were lower than in deeper tailings for Zn, Mn, Ni, and Co (Figure S12), mass transfer calculations revealed surficial depletion (−τ) in the oxic surface zone corresponding with enrichment (+τ) at depth (40–90 cm) for Zn, Mn, Ni, Cu, and Co (Figure S13).

**Figure 3.**
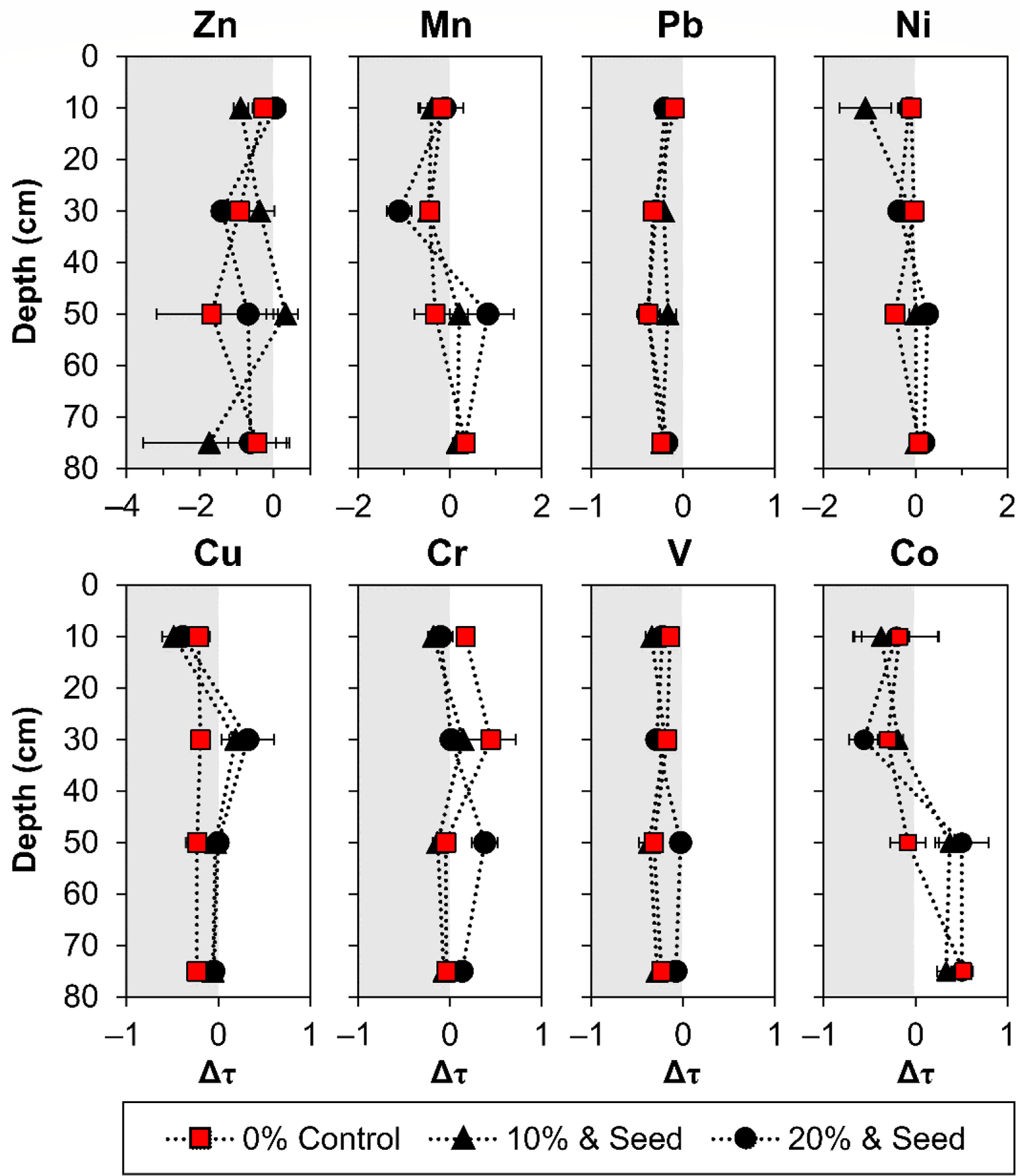
Mass transfer dynamics in field cores. The change in enrichment/depletion (Δτ) of each metal from the beginning to end of phytostabilization are shown for the control and seeded 10% and 20% plots. Error bars show standard deviation among samples collected from quadruplicate field plots, *n* = 4. Depth is plotted as the mid-point of each homogenized core depth increment.

The temporal change in enrichment/depletion (Δτ), based on the τ value at the beginning and the end of the 3y experiment, revealed that the depletion of Mn and Co occurred from 0 to 20 cm and 20 to 40 cm during phytostabilization (Figure 3). The concurrent enrichment of Mn and Co was observed at 40–60 cm and 60–90 cm, indicating the downward movement of Mn and Co from the near surface to depths (Figure 3). Compost addition and the establishment of vegetation enhanced Mn, Ni, Cu, Cr, and Co translocation to depths, observed as a −Δτ in the near surface and +Δτ at depths compared to the control. Metals Pb and V showed low variability in both the total concentration and lability among all treatments (Figure 3). For the labile metals, a greater change toward depletion (−Δτ) over time was observed above 40 cm, while a greater change toward enrichment (+Δτ) was observed below 40 cm depth in compost treatments compared to the control after 3 y. Zinc was the only metal that exhibited a treatment Δτ greater than the control at the surface 0–20 cm in the 20% seeded amendment (Figure 3). Copper exhibited treatment enrichment over time at the 20–40 cm depth, while chromium was enriched for the control (Figure 3).

## 4. Discussion

This experiment investigated field-scale, direct-planting, compost-assisted phytostabilization in legacy acid sulfide tailings undergoing surficial oxidative weathering in a (semi-) arid environment. The temporal changes in metal lability and translocation to depth investigated at four time points over three years with four depth increments from 0 to 90 cm showed dynamic responses to remediation. Despite the variable solubilities and geochemical properties of the metals considered (V, Cr, Mn, Co, Ni, Cu, Zn, and Pb), it was hypothesized that secondary minerals formed during oxidative weathering (e.g., (oxyhydr)oxides and sulfates) would contribute to the overall attenuation of metals. It was expected that the abundance of secondary minerals in the surficial tailings above 20 cm and the near-neutral pH below 20 cm depth would lead to metal sequestration in the near surface with little translocation to the mostly unweathered 60–90 cm depth increment (Hayes et al., 2014; Root et al., 2015). The results show that 3 y phytostabilization treatments affect metal lability and mobility greatest at the surface 0–20 cm. Continued metal enrichment at intermediate depths (20–40 cm) after the establishment of vegetation indicates that evapotranspiration in the (semi-) arid climate does not prevent the downward flux of solution-phase-dissolved or colloidal metals, as has been shown to occur in hyper-arid climates [44], Although metals migrated from the top 20 cm to the horizon below, this extended field study demonstrated that metals are retained in the rhizosphere, as hypothesized, under OM and planting treatments. However, the final-year development of enrichment at 60–90 cm for Cr and Mn and depletion at shallow depths for V, Cu, Zn, and Pb indicate that further metal translocation with increased hydrologie throughflux may be a long-term concern.

### 4.1. Acidification

The progressive acidification of amended tailings over the three-year study indicated that the initial pH buffering via OM (manure compost pH = 10.0 ± 0.1) was temporary, and tailings acidification progressed despite the establishment of vegetation. The temporary neutralization in the tailings via compost addition was not sufficient to prevent acid-mediated metal dissolution [45,46]. A Pearson’s correlation coefficient matrix of pH compared to the extractable metals showed the relationships between the highly labile water-soluble pools and acidity (Table 2). Correlation constants at the 95% confidence interval from averaged quadruplicate samples of 0% compost control, 10% compost and seed, 20% compost, and 20% compost and seed for all depths and years were compared (*n* = 64 averaged values) (Table 2). The correlation coefficients revealed a strong (negative) relationship between tailings pH and lability. Water-soluble metals that form carbonates and sulfate salts (Mn, Co, Ni, Cu, and Zn) showed higher solubility at lower pH (Table 2). It is well-documented that increasing pH plays an important role in decreasing the solubility of metal cations and adsorption to (oxyhydr)oxide and humic surfaces through surface functional group deprotonation [47,48]. Acidity showed very little correlation to the water solubility of V, Cr, or Pb, although the water-soluble pools of V and Cr were strongly correlated to each other (Table 2). Metal lability in other systems of weathering mine tailings have shown relatively high solubility for Mn and Zn and low solubility of Pb under similar, but unamended, field conditions [9,45,49]. The stabilization of lead in acid mine tailings via incorporation into jarosite likely prevents lability, even under changing pH or organic matter additions [50].

### 4.2. Metal Lability

Enrichment was observed for Mn, Co, Ni, Cu, and Zn (Figures S12 and S13) at >20 cm compared to the near surface. This is attributed to surficial mobilization (and depletion) with deposition (and enrichment) at depths due to downward translocation in response to irrigation. The release of these metals in the first steps of the sequential extraction showed higher surface lability that progressively decreased with depth. Temporal enrichment analysis (Δτ) confirmed that downward mobilization was taking place and was enhanced by phytostabilization (Figure 3). Mass transfer from the surface to depths in the tailings was especially well-defined for Mn, Co, and Zn enrichment/depletion (τ) (Figure S13) and dynamic mass transfer (Δτ) calculations (Figure 3).

**Figure 4.**
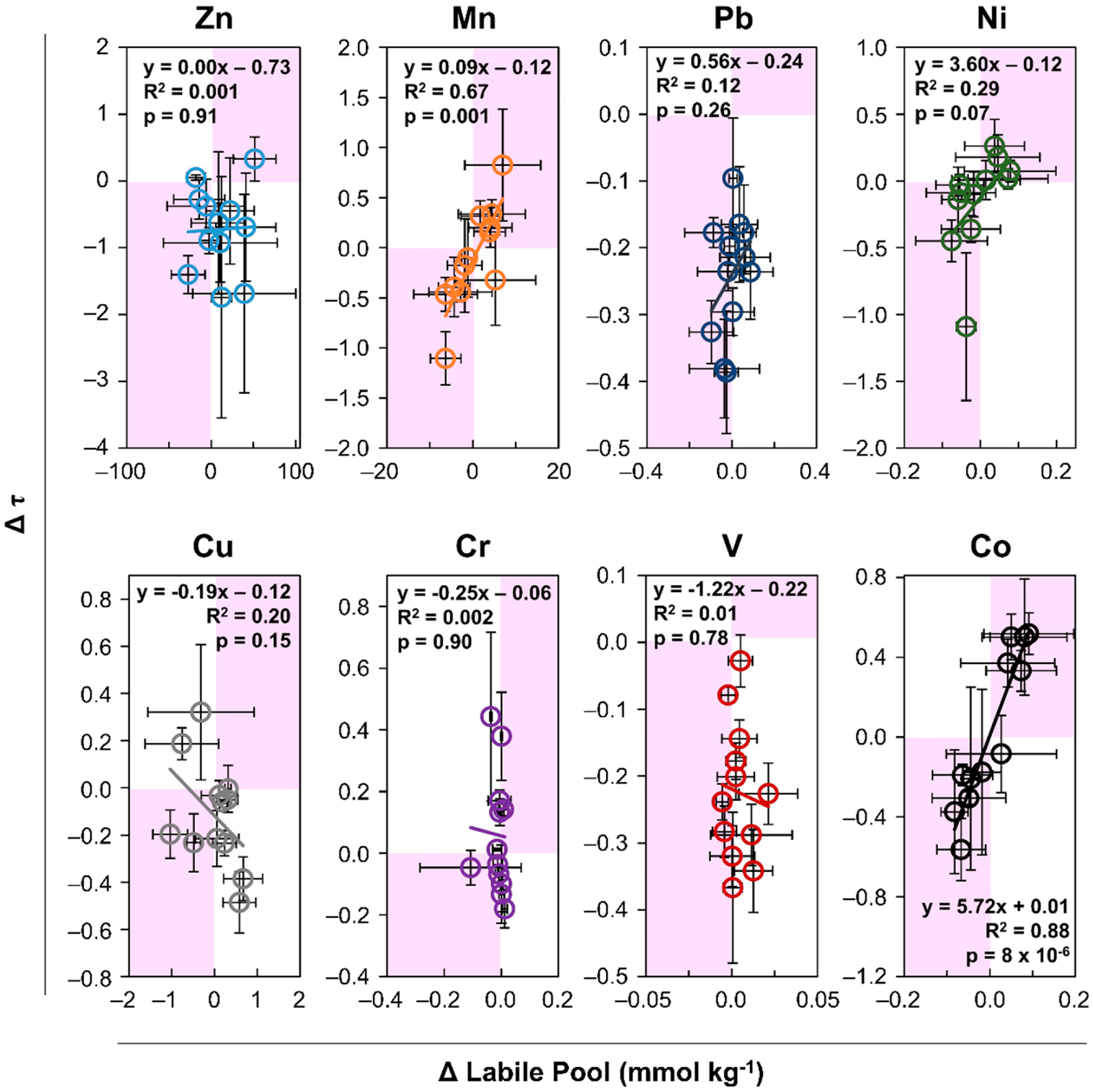
Temporal relationship between labile pool and enrichment/depletion. The relationship between the change over 3 years in the labile pool (sum of water soluble and adsorbed) and change in enrichment/depletion (Δτ) is shown for depth increments 0–20, 20–40, 40–60, and 60–90 cm for 0% compost control, 10% compost and seed, and 20% compost and seed treatments. Error bars show standard deviation among samples collected from quadruplicate field plots, *n* = 4 for each depth and treatment. The statistical parameter R^2^ indicates goodness of fit of a linear relationship according to the equation shown describing the linear best fit. A statistical parameter *p*-value < 0.05 describes a statistically significant linear slope reported for a 95% confidence interval (*α* = 0.05, *n* = 12, for a two-tailed test).

**Figure 5.**
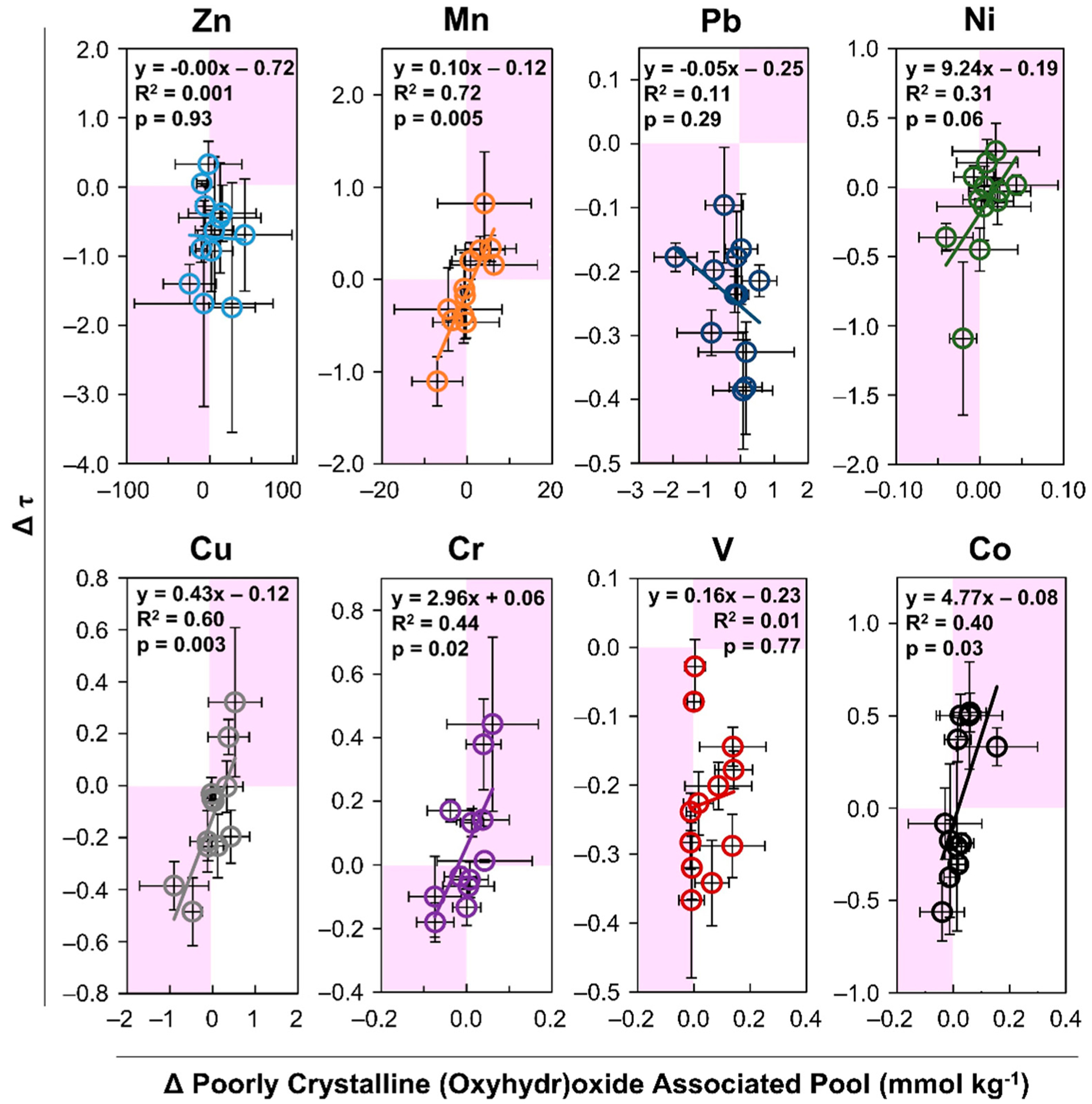
Temporal relationship between recalcitrant (oxyhydr)oxide-associated metals and enrichment/depletion. The relationship between the change in metal concentration over 3 years from poorly crystalline (oxyhydr)oxide-associated pools and change in enrichment/depletion (Δτ) for depth increments 0–20, 20–40, 40–60, and 60–90 cm for 0% compost control, 10% compost and seed, and 20% compost and seed treatments. Error bars show standard deviation from quadruplicate field plots, *n* = 4 for each depth and treatment. The statistical parameter R^2^ indicates goodness of fit of a linear relationship according to the equation shown describing the best fit. A statistical parameter *p*-value < 0.05 describes a statistically significant linear slope reported for a 95% confidence interval (*α* = 0.05, *n* = 12, for a two-tailed test).

Regression analysis was employed to investigate a statistically significant (*p* < 0.05) linear relationship between Δτ and Δ lability at the 95% confidence interval for a two-tailed test. The relationship between lability and mobility over time was examined first by comparing the change in lability measured as the change in the labile pool concentration, versus the change in enrichment during the three-year study (Figure 4). Second, the change in the recalcitrant but extractable pool of metals associated with poorly crystalline (oxyhydr)oxide secondary minerals versus the change in enrichment during the three-year study was analyzed (Figure 5). The change over 3y of averaged field quadruplicates for all four depth increments of 0% control, 10% compost and seed, and 20% compost and seed (*n* = 12) showed a significant relationship for the easily mobilized pools of Cr and Mn (Figure 4). Among the metals mobilized in the labile fraction and the recalcitrant fraction, only Mn and Co exhibited a statistically significant linear relationship between temporal changes in lability and enrichment (Figure 4). These results support the observation that Mn and Co exhibit the greatest mobilization compared to other metals and suggests that in the oxidative weathering environment, Mn and Co are the most susceptible to water solubility. Interestingly, there was no significant relationship between Δτ and Δ lability for Zn, Pb, Ni, Cu, Cr, or V. The relation between change in τ and lability over time was positive (slope) for Mn, Pb, Ni, and Co, but only significant at the 95% confidence interval for Mn and Co. A negative relationship was observed for Cu, Cr, and V. While Zn lability was strongly correlated with acidity, it showed no temporal relationship with enrichment or depletion.

Evidence of a significant relationship between the change in enrichment and changes in the poorly crystalline (oxyhydr)oxide-associated Mn, Cu, Cr, and Co supports the hypothesis of secondary precipitates attenuating metals (Figure 5). This observation is consistent with metal sequestration in other tailing systems [45,51], Comparing dynamic elemental enrichment/depletion and extractability over 3 y reveals that Mn and Co were most susceptible to mobilization from the tailings surface. Significant trends (*p* < 0.05) in surficial depletion (and enrichment at depths) correlating to a change in extractability show that Mn and Co are leached from the near surface and deposited at greater depths. Copper in the near-surface oxic tailings showed increased lability and translocation to depths after phytostabilization, indicating that weathering and OM enhance copper mobility The zinc mobilized from the surface tailings was likely the divalent cation, released from sulfate salts and sorption sites on (oxyhydr)oxides at the start of this experiment. These sulfates and (oxyhydr)oxides are weathering products of the original zinc mineral sphalerite (ZnS) and secondary mineral smithsonite (ZnCO_3_) [17,39]. Zinc lability correlated with decreasing pH. The greatest enrichment of zinc occurred at 40–60 cm in the control plots, indicating that phytostabilization decreased zinc mobility in the oxic zone of the tailings. Although Zn was observed to translocate from surface tailings to depths, a relationship with the operationally defined sequential extraction pools was not observed.

### 4.3. Recalcitrant Metals

Lead, vanadium, and chromium were generally recalcitrant in the tailings and were only extracted at appreciable fractions from the pool associated with poorly crystalline (oxyhydr)oxide minerals. In a similar fashion, all three elements exhibited a lack of correlation between mobilization and pH (Table 2). Vanadium and chromium likely originated as minor components in silicates, sulfides, and carbonates. Lead in deep tailings was predominantly in the form of the sulfide galena and carbonate cerussite (PbCO_3_) [17,39]. Lead in the surface tailings mainly existed as plumbojarosite [17]. Although the lead mineralogy of the surface tailings differed significantly from the deep tailings, there was little difference in lead lability or mobility throughout the depths considered. This indicates that in the IKMHSS tailings, weathering and phytostabilization do not increase lead mobility. Similarly, vanadium exhibits both low lability and low mobilization with respect to treatment, time, and depth, generally being unaffected by oxidative weathering and phytostabilization. Although chromium exhibited high recalcitrance for all treatments and depths throughout the three-year study, translocation to depths was observed for composted treatments during phytostabilization and showed a statistically significant linear relationship with the pool associated with poorly crystalline (oxyhydr)oxides. Unlike in lead and vanadium, chromium release to porewaters and the promotion of translocation was enhanced during phytostabilization.

## 5. Conclusions

Although compost-assisted phytostabilization initially raised the tailings’ pH, oxidative weathering and acid generation potential in surface tailings produced progressive acidification which was the primary mechanism for the increased lability and translocation of metals over time. The mobilization of some metals across short distances (<1 m) from the surface to depths occurred in all treatments, even in phytostabilized plots that had an initial pH neutralization and buffering provided by OM addition. The establishment of a vegetation cap or the addition of compost may have contributed to an enhanced rate of metal translocation, especially for Mn and Co, relative to the irrigation-only control. However, sequential extraction analysis did not indicate that organic matter or vegetation influenced a change in the specific sequential extraction-assigned speciation pools for any of the metals investigated. Further, the magnitude of 1D translocation to depth was low, generally less than 20 cm over 3 y. While Pb and V remained largely recalcitrant, Mn, Cu, Cr, and Cu fate and mobility were governed by the stability of poorly crystalline (oxyhydr)oxide secondary mineral precipitates forming under oxidative conditions. Although Zn and Ni were shown to translocate to depths over time, there was not a significant relationship between mobilization and the labile pools considered. These results highlight the importance of considering both depth and time in assessing of the utility and success of phytostabilization for sequestering metal contaminants in near-surface mine tailings.

## Supplementary Material

SI

## Figures and Tables

**Table 1.** Specific sequential extraction steps for mine tailing samples.

Step	Extractant	Conc. (M)	Time, Temp.	Target Phase	References
1	DI H_2_O, Milli-Q water (18.2 MΩ-cm)	–	1 h, 25 °C	Soluble salts	[41]
2	NaH_2_PO_4_, monosodium phosphate, pH 5	1	24 h, 25 °C	Exchangeable and adsorbed ions	[42]
3	Tamm’s reagent, ammonium oxalate, pH 3, dark	0.2	2 h, 25 °C	Poorly crystalline Al, Mn, and Fe (hydr)oxides	[41,42]

**Table 2.** Correlation among water-extractable fractions for different elements and pHs. Pearson’s linear correlation coefficient matrix (*p* < 0.05) was calculated for the percent of water-extractable elemental components with respect to microwave-assisted aqua regia digestible total concentration for averaged quadruplicate IKMHSS tailings samples from all treatments, depths, and years (*n* = 64).

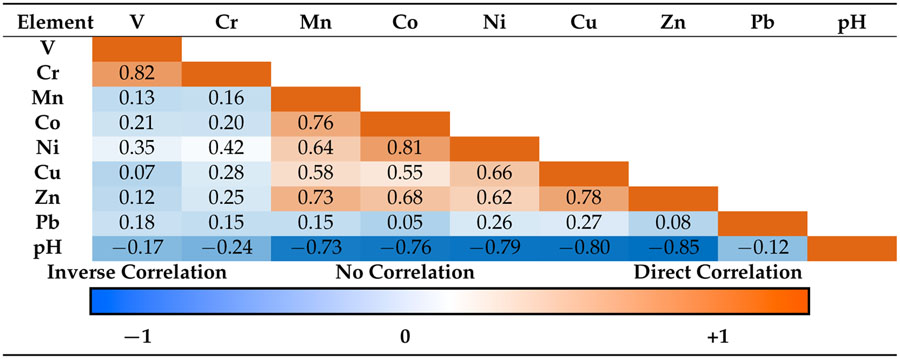

## Data Availability

Data is contained within the article or supplementary material.

## Highlights

- The acidification of oxidizing surface tailings largely controlled the subsurface fate of Mn, Co, Ni, Cu, and Zn.
- Mass transfer was observed for phytostabilization treatments and the control for Mn, Co, Cu, and Zn translocation from the surface.
- Phytostabilization promoted Cr mobility from the surface and enrichment at depths relative to the unamended control.
- Phytostabilization reduced zinc mobility in the oxic zone.
- Pb was mostly recalcitrant as plumbojarosite and was not mobilized by weathering or compost-assisted phytostabilization.
