# Supplementary material for: Metal Lability and Mass Transfer Response to Direct-Planting Phytostabilization of Pyritic Mine Tailings
Source: Minerals (Basel). Author manuscript; Available in PMC 2022 Nov 22. (PMC9681136; doi:10.3390/min12060757)
Supplement: SI [file NIHMS1848878-supplement-SI.pdf]

Article

Supplementary Materials: Metal Lability and Mass Transfer Response to Direct-Planting Phytostabilization of Pyritic Mine Tailings

Corin M. Hammond, Robert A. Root *, Raina M. Maier and Jon Chorover

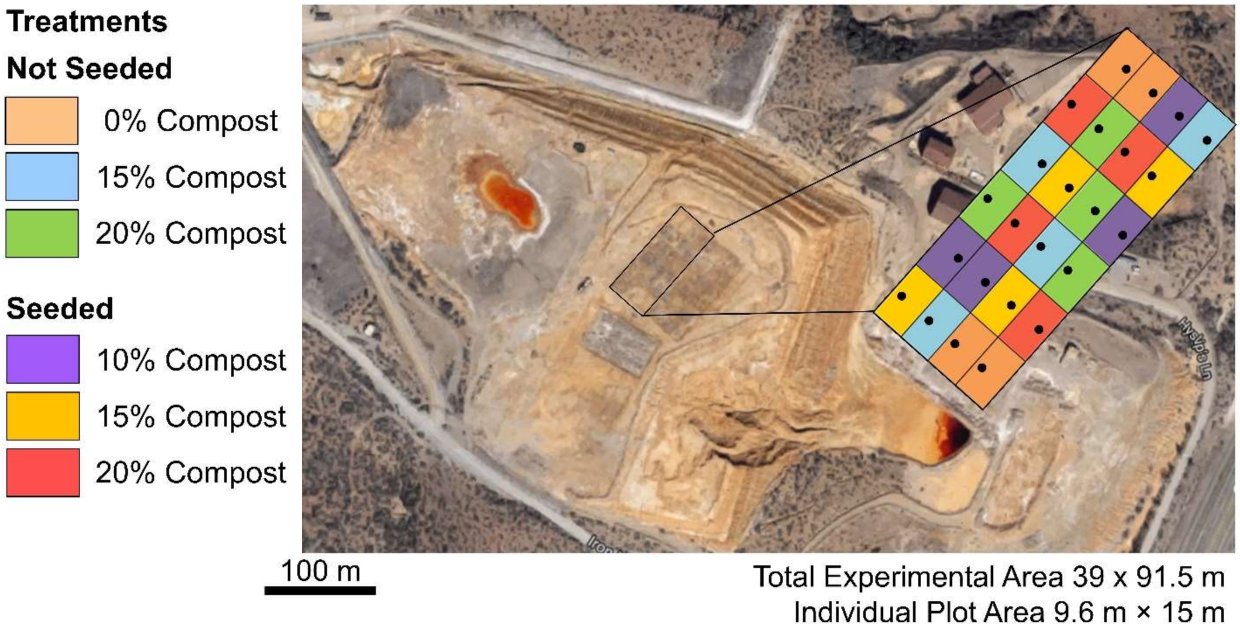

Figure S1. Aerial view of the IKMHSS tailings phytostabilization study field site. The black square delineates the study area. The inset shows a diagram of the 24 plots color coded according to treatment. Black dots denote core locations that are located central along the long axis but actually varied along the short axis of each plot annually. Variation in coring location was essential to collect samples at a distance from previous core holes to avoid sample contamination by non-treatment related oxidative weathering by increasing oxygen diffusion to deep tailings.

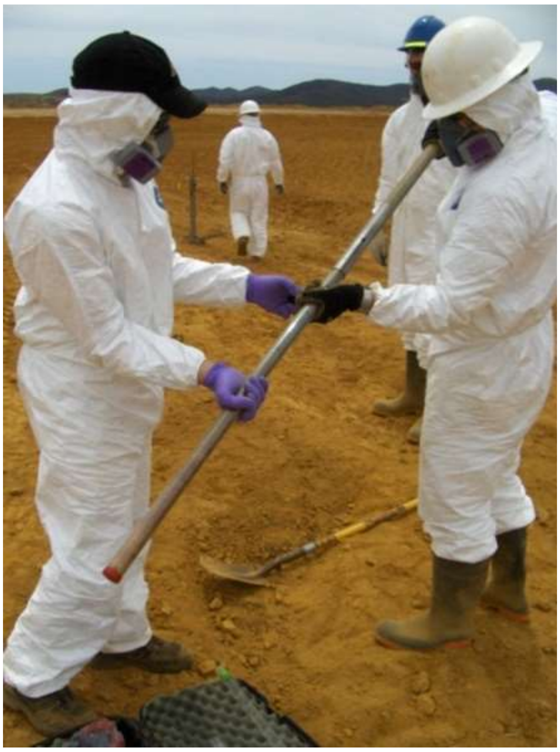

Figure S2. Core samples collected annually. IKMHSS tailings cores (2.54 cm diameter, up to 91.44 cm length) were collected for each field plot annually between May–July in 2010 (time zero), 2011, 2012, and 2013.

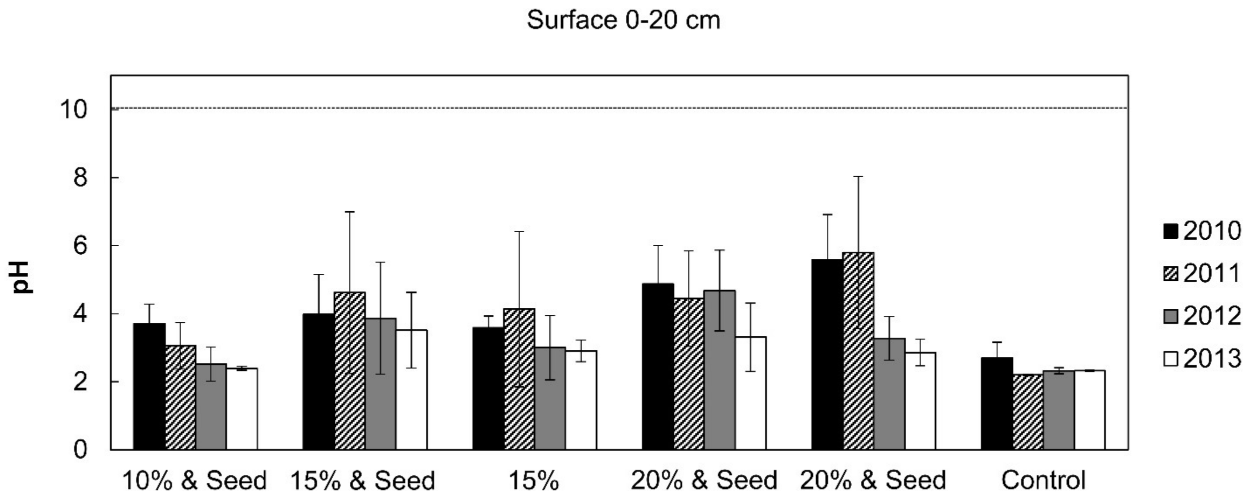

Figure S3. Surface IKMHSS tailings pH measured from a slurry. Surficial (0–20 cm depth) pH measurements of six tailings treatments with respect to time measured from a 1:1 tailings: DI H_2O mixture. Horizontal line shows the pH of the dairy compost used for amendment in treatments.

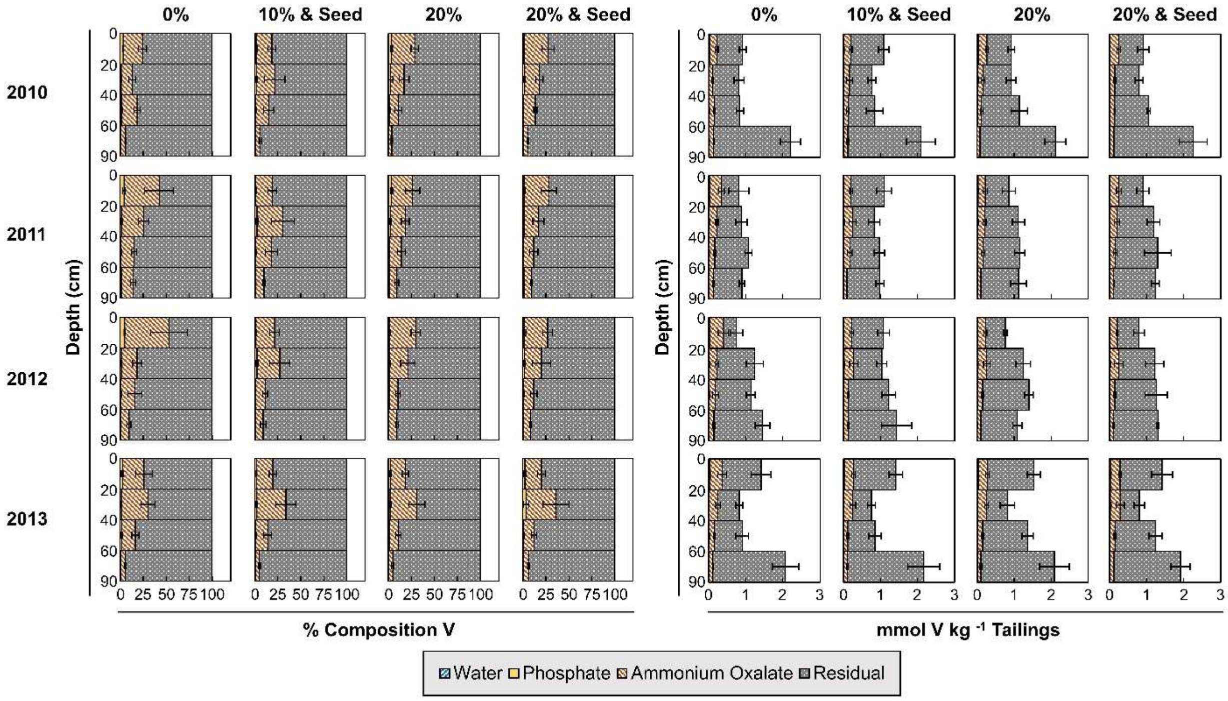

Figure S4. Vanadium sequential extraction results. Fraction of vanadium that is solubilized during each step of the sequential extraction. Percent extractable is relative to aqua regia digestible totals reported in Table S4. Error bars denote standard deviation among quadruplicate field replicates (n = 4).

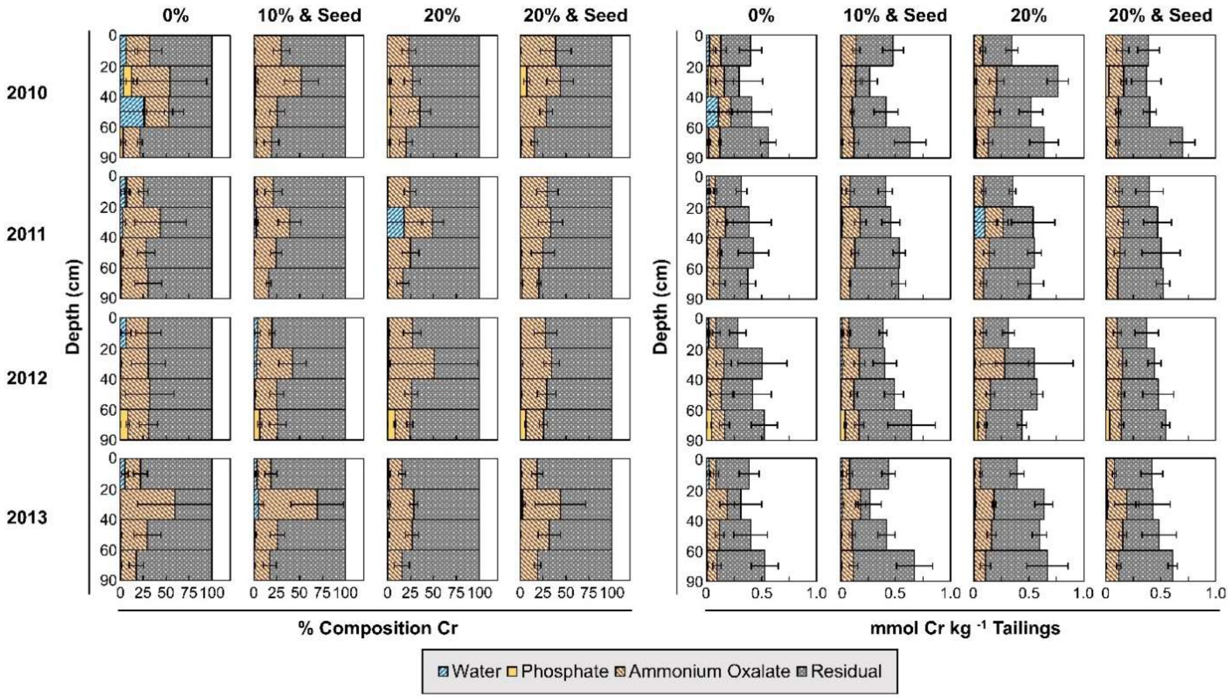

Figure S5. Chromium sequential extraction results. Fraction of chromium that is solubilized during each step of the sequential extraction. Percent extractable is relative to aqua regia digestible totals reported in Table S5. Error bars denote standard deviation among quadruplicate field replicates (n = 4).

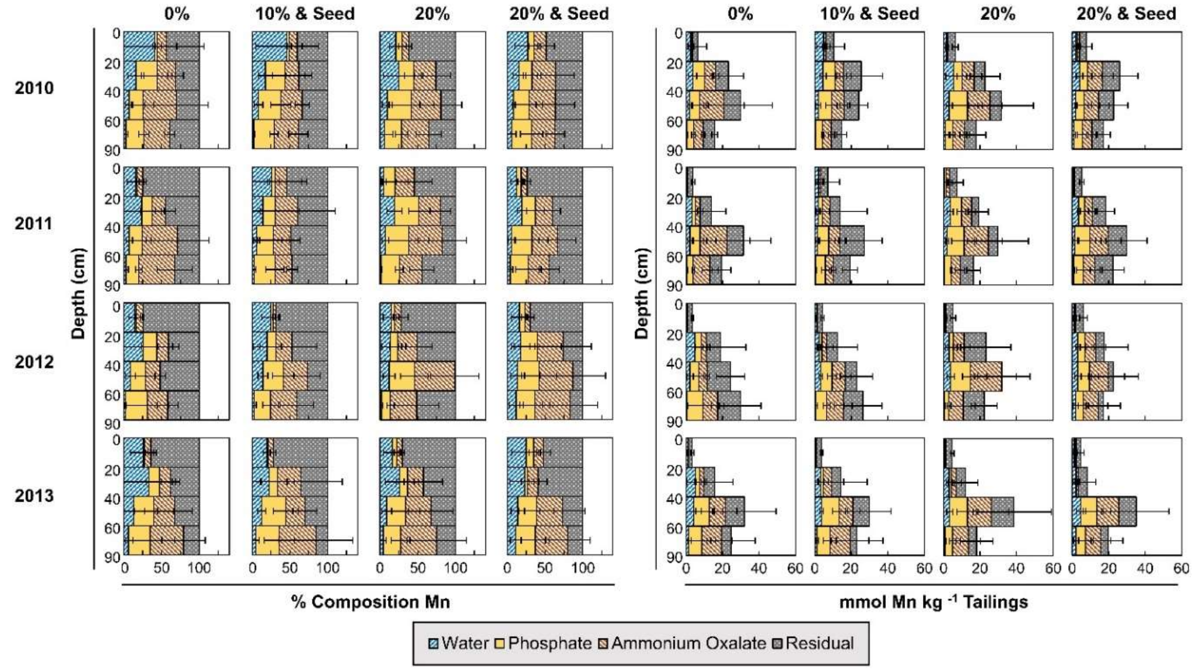

Figure S6. Manganese sequential extraction results. Fraction of manganese that is solubilized during each step of the sequential extraction. Percent extractable is relative to aqua regia digestible totals reported in Table S6. Error bars denote standard deviation among quadruplicate field replicates (n = 4).

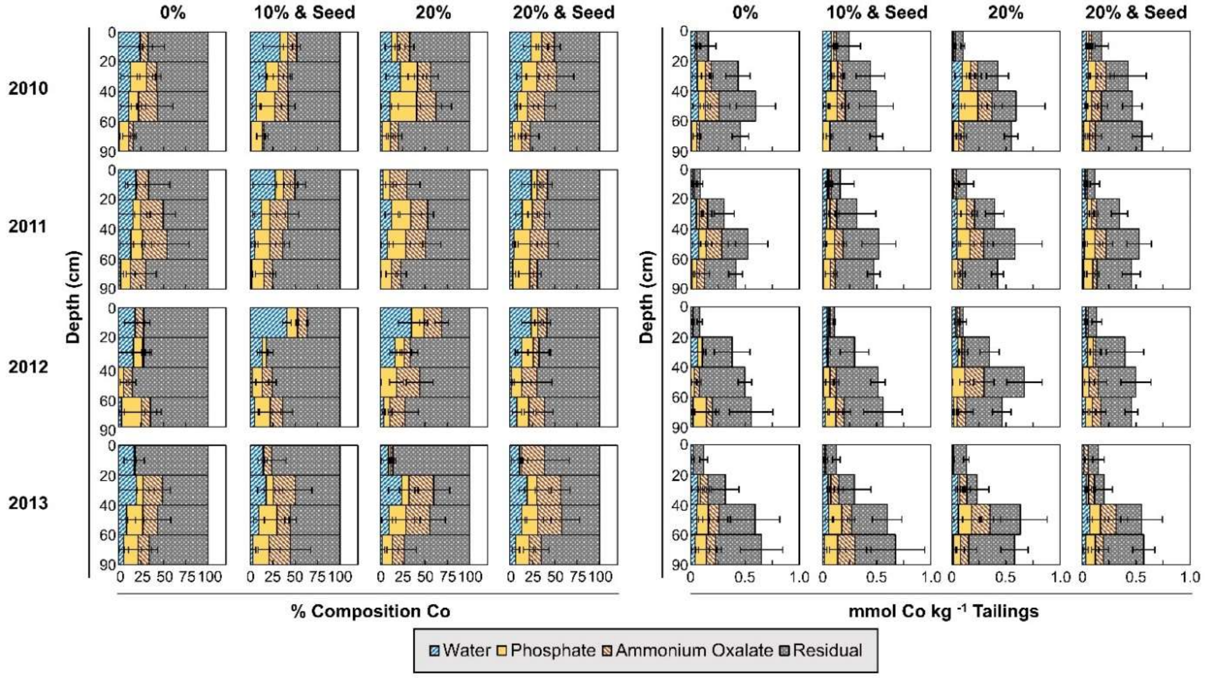

Figure S7. Cobalt sequential extraction results. Fraction of cobalt that is solubilized during each step of the sequential extraction. Percent extractable is relative to aqua regia digestible totals reported in Table S8. Error bars denote standard deviation among quadruplicate field replicates (n = 4).

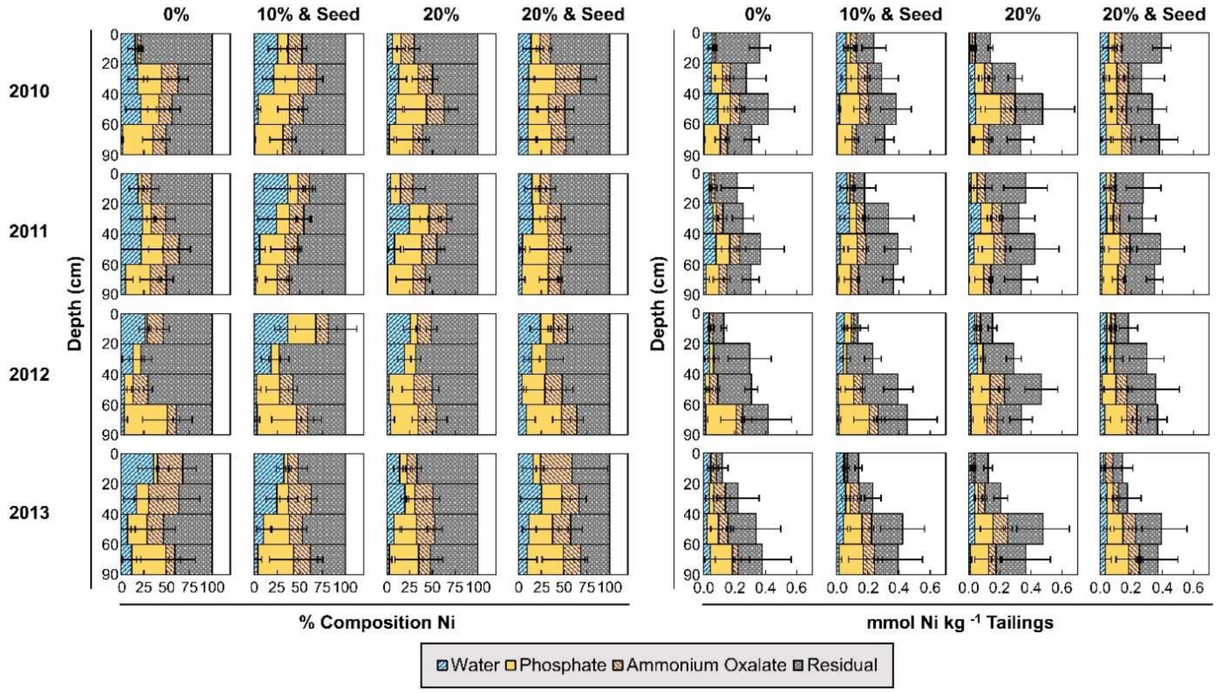

Figure S8. Nickel sequential extraction results. Fraction of nickel that is solubilized during each step of the sequential extraction. Percent extractable is relative to aqua regia digestible totals reported in Table S9. Error bars denote standard deviation among quadruplicate field replicates (n = 4).

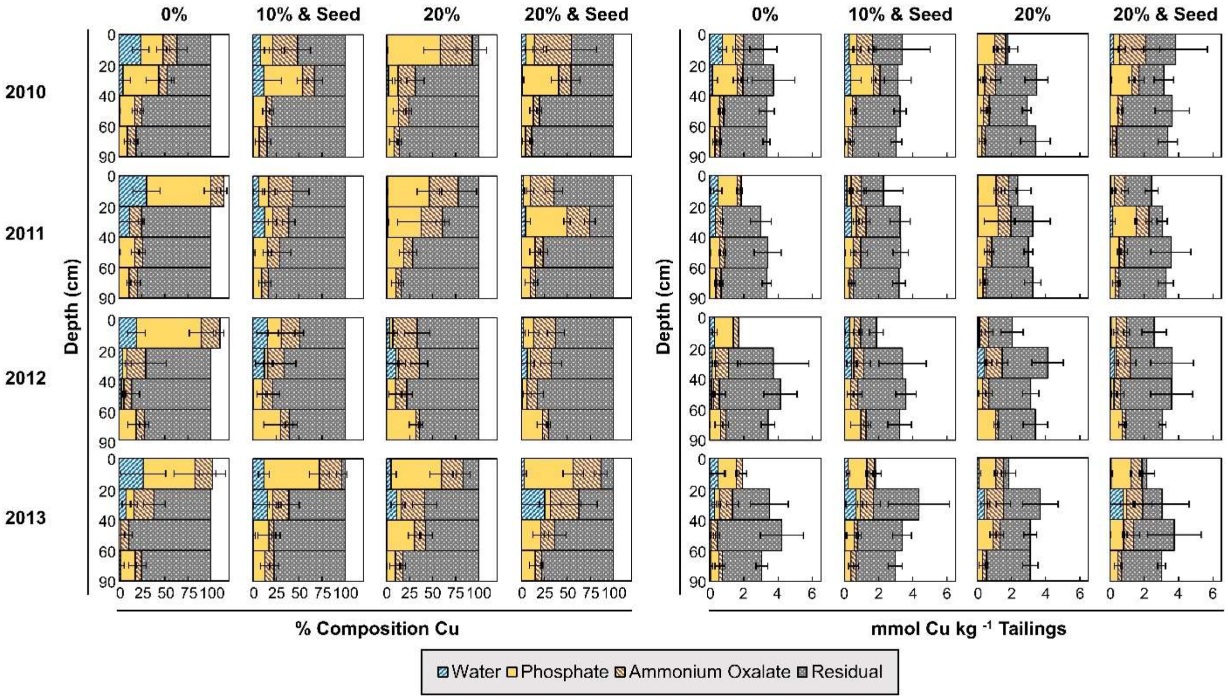

Figure S9. Copper sequential extraction results. Fraction of copper that is solubilized during each step of the sequential extraction. Percent extractable is relative to aqua regia digestible totals reported in Table S10. Error bars denote standard deviation among quadruplicate field replicates (n = 4).

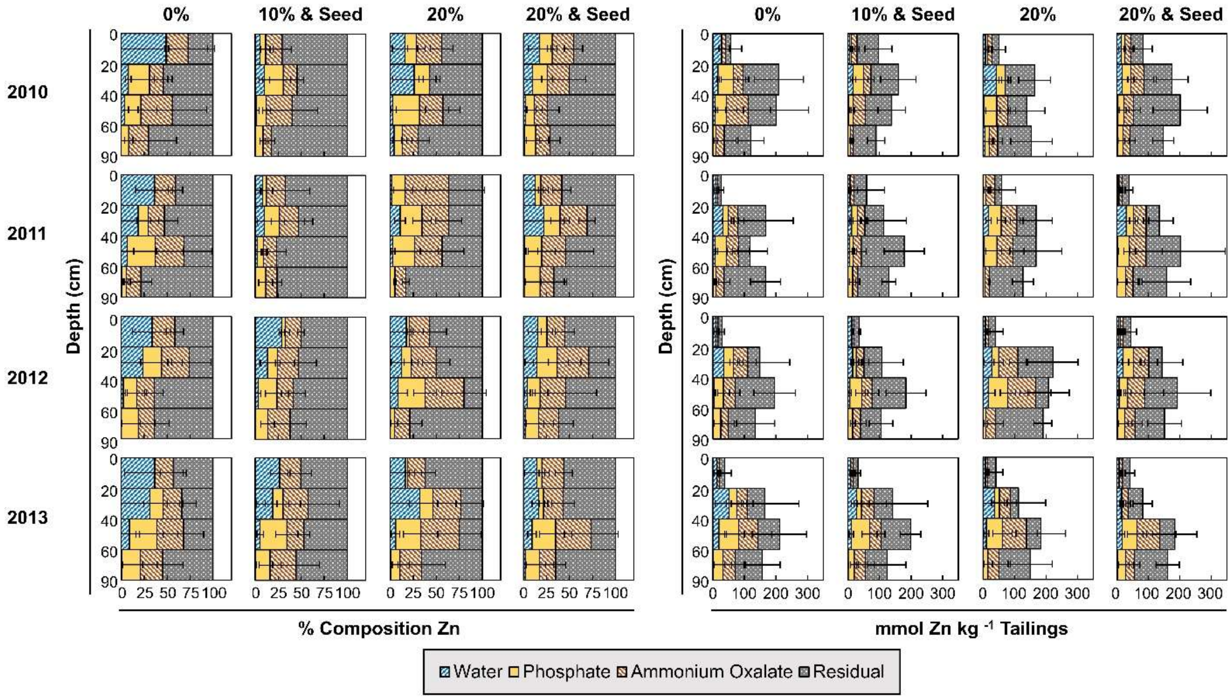

Figure S10. Zinc sequential extraction results. Fraction of zinc that is solubilized during each step of the sequential extraction. Percent extractable is relative to aqua regia digestible totals reported in Table S11. Error bars denote standard deviation among quadruplicate field replicates (n = 4).

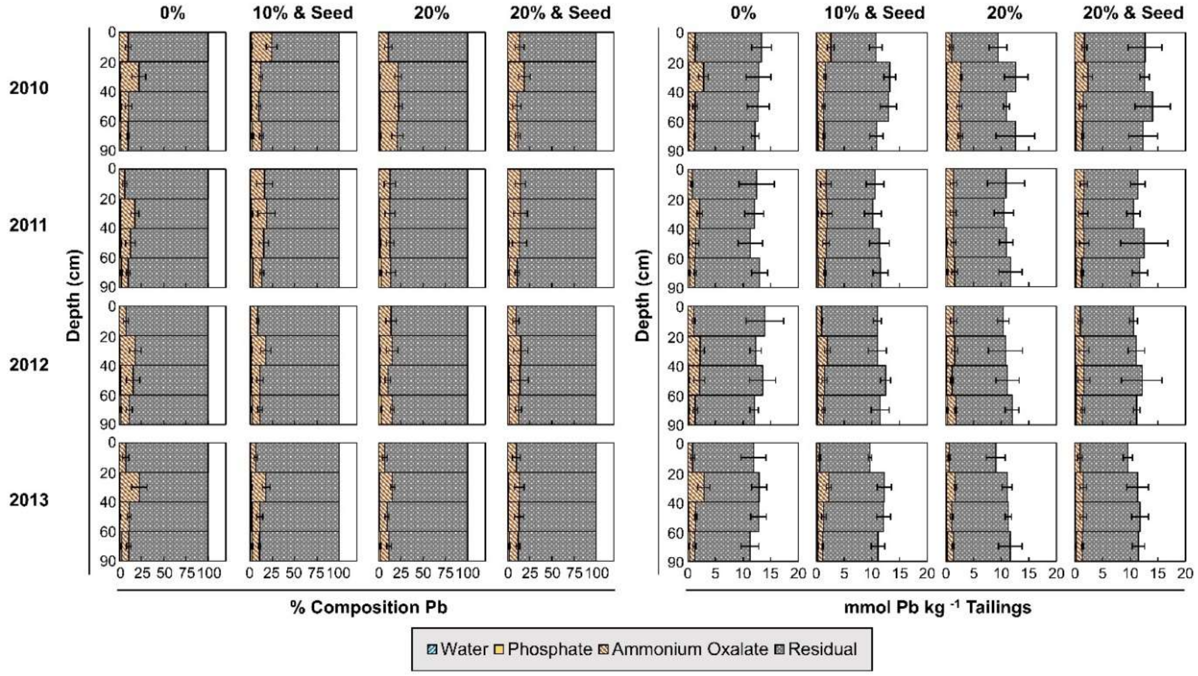

Figure S11. Lead sequential extraction results. Fraction of lead that is solubilized during each step of the sequential extraction. Percent extractable is relative to aqua regia digestible totals reported in Table S13. Error bars denote standard deviation among quadruplicate field replicates (n = 4).

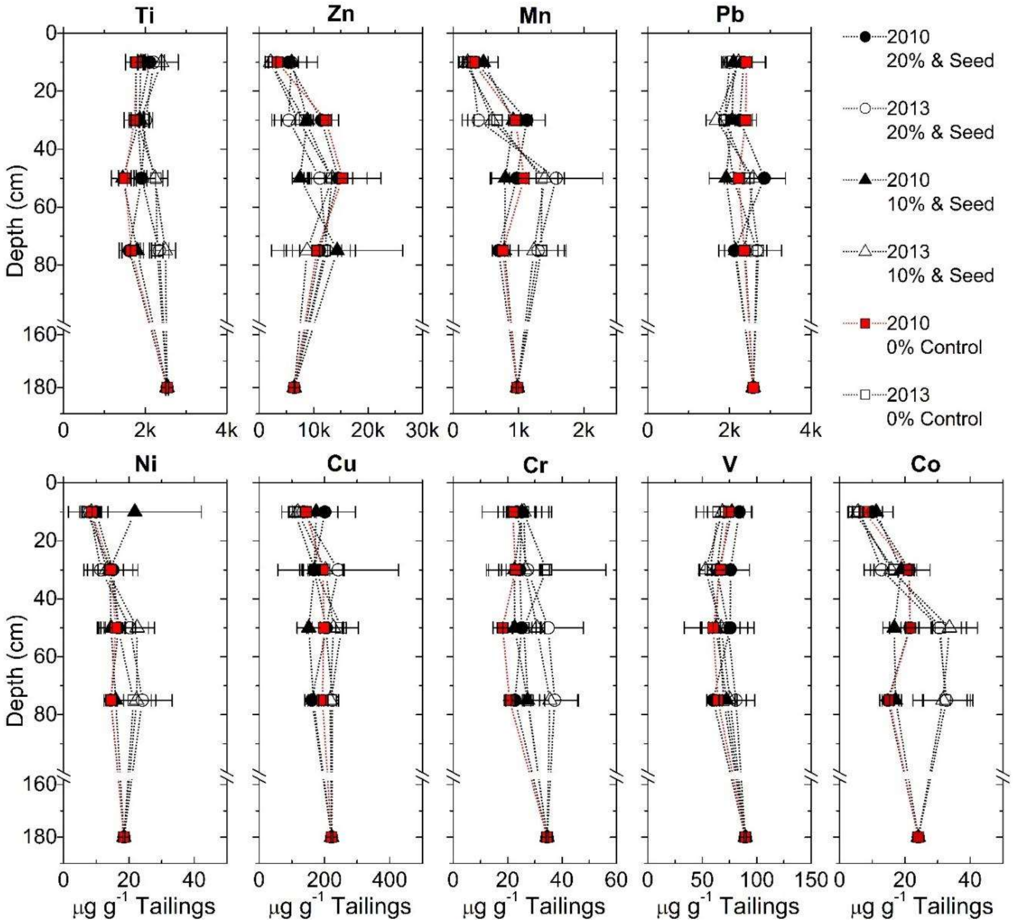

Figure S12. Total elemental concentration by microwave-assisted HF digestion. Concentration values were used to calculate enrichment/depletion plots (Figure 2) with respect to unweathered parent material collected at 180 cm depth. Error bars denote standard deviation for n = 4 field replicates. Data points of the time zero (2010) control are shown in red to highlight deviation of concentration with respect to treatment and time.

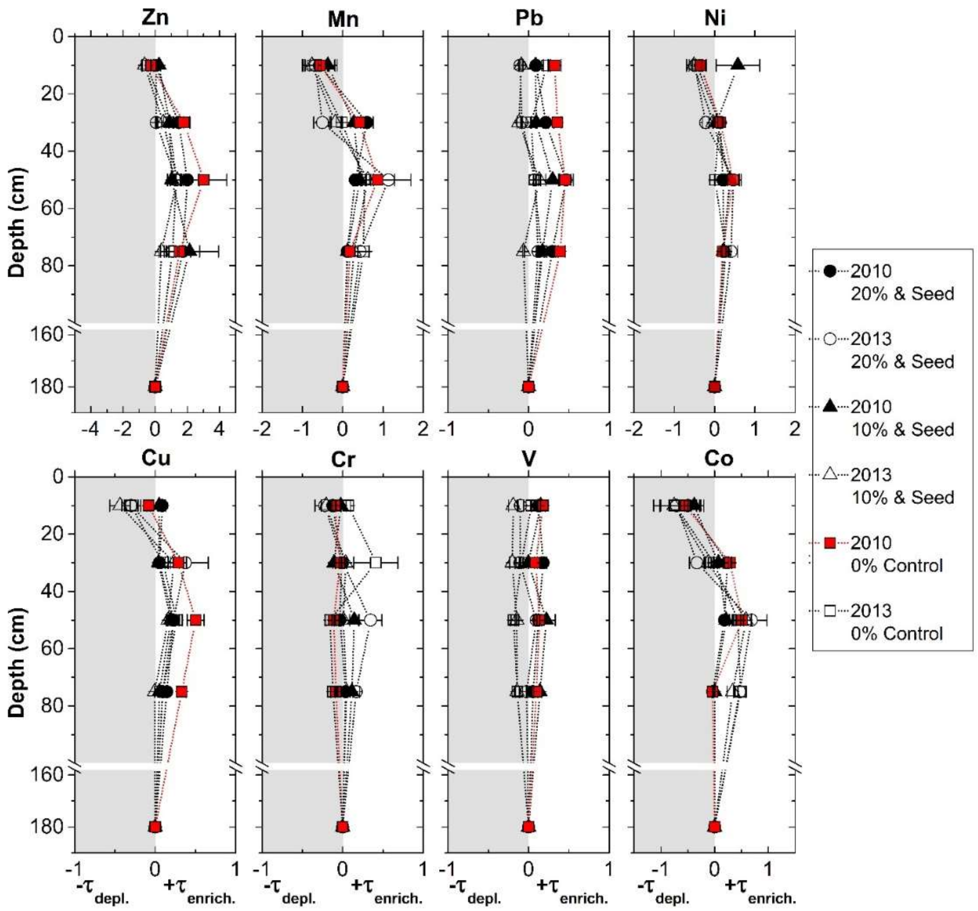

Figure S13. Elemental tau (τ) plots of enrichment/depletion with respect to unweathered parent material collected at 180 cm depth. Data points of the time zero (2010) control are shown in red to highlight deviation of concentration with respect to treatment and time. Depth is the average of each previously defined homogenized core depth increment. τ plots show Ti normalized Zn, Mn. Pb, Ni, Cu, Cr, V, and Co concentrations with depth relative to unweathered deposited material from 180–183 cm (see Eq. 1). The shaded region shows relative depletion (−τ) while the white region shows relative enrichment (+τ). Error was propagated from standard deviation values (n = 4 field replicates) for all four sample depths. For the parent material sample n = 1.

Table S1. Tailings pH from a 1:100 solid to solution mixture. Changes in final pH from the first step (deionized water, 1h, rt) of the sequential extraction procedure are reported for IKMHSS tailings samples with respect to treatment and time. Standard deviation for n = 4 field replicates is reported in parentheses.

Treatment	Depth (cm)[b]	pH[a] 2010	pH[a] 2011	pH[a] 2012	pH[a] 2013
0%	0–20	3.0 (0.4)	2.8 (0.1)	2.8 (0.1)	2.7 (0.1)
0%	20–40	6.1 (1.2)	4.2 (1.3)	4.6 (0.9)	4.4 (0.8)
0%	40–60	6.3 (1.1)	6.2 (1.1)	6.3 (1.1)	6.9 (0.3)
0%	60–90	7.0 (0.3)	7.0 (0.5)	7.1 (0.2)	7.0 (0.4)
10% & Seed	0–20	4.3 (0.4)	3.8 (0.7)	3.3 (0.6)	2.8 (0.2)
10% & Seed	20–40	5.2 (1.7)	4.6 (1.8)	4.3 (1.6)	4.1 (1.6)
10% & Seed	40–60	7.0 (0.5)	6.5 (0.4)	7.1 (0.2)	7.0 (0.2)
10% & Seed	60–90	7.4 (0.2)	7.2 (0.2)	7.2 (0.4)	7.4 (0.3)
20%	0–20	4.7 (0.8)	5.4 (1.5)	3.8 (0.4)	3.3 (0.5)
20%	20–40	5.9 (1.0)	6.1 (0.4)	5.0 (1.2)	3.5 (1.0)
20%	40–60	6.8 (0.2)	6.9 (0.1)	6.8 (0.4)	7.0 (0.1)
20%	60–90	6.8 (0.2)	7.1 (0.0)	7.1 (0.1)	7.2 (0.3)
20% & Seed	0–20	4.7 (0.5)	4.4 (0.7)	4.4 (0.5)	4.1 (0.7)
20% & Seed	20–40	5.9 (0.9)	5.4 (1.2)	5.3 (1.5)	4.0 (0.8)
20% & Seed	40–60	6.5 (0.5)	6.8 (0.3)	6.8 (0.7)	6.8 (0.4)
20% & Seed	60–90	7.3 (0.1)	6.9 (0.1)	6.8 (0.2)	7.0 (0.2)

[a] pH was measured on water extractions (sequential extraction step 1, 1:100 solid: DI H_2O mass ratio)
[b] Depth is the composited interval below the surface.

Table S2. Aqua regia extractable aluminum. Microwave-assisted aqua regia digestible aluminum concentrations are reported for the IKMHSS tailings samples with respect to treatment and time.

Treatment	Depth[b] (cm)	Al Concentration (mmol kg^{-1})[a] 2010	2011	2012	2013
0%	0–20	450 (40)	400 (90)	360 (10)	360 (50)
0%	20–40	600 (140)	420 (70)	790 (240)	630 (100)
0%	40–60	490 (60)	440 (60)	630 (190)	530 (150)
0%	60–90	550 (50)	460 (110)	700 (100)	560 (110)
10% & Seed	0–20	540 (190)	410 (130)	370 (60)	370 (100)
10% & Seed	20–40	570 (40)	520 (50)	490 (50)	630 (70)
10% & Seed	40–60	400 (90)	570 (40)	490 (70)	410 (90)
10% & Seed	60–90	570 (130)	570 (60)	710 (300)	560 (140)
20%	0–20	490 (80)	510 (30)	440 (60)	380 (70)
20%	20–40	430 (40)	610 (140)	620 (150)	360 (160)
20%	40–60	540 (60)	570 (40)	540 (50)	560 (40)
20%	60–90	560 (90)	550 (110)	470 (80)	560 (130)
20% & Seed	0–20	510 (110)	510 (130)	550 (180)	380 (100)
20% & Seed	20–40	620 (80)	620 (180)	560 (100)	570 (110)
20% & Seed	40–60	390 (30)	680 (170)	470 (60)	490 (50)
20% & Seed	60–90	630 (130)	610 (100)	610 (5)	530 (60)

[a] Extraction performed according to EPA method 3051A. Standard deviation for n = 4 field replicates reported in parentheses.
[b] Depth is the composited interval below the surface.

Table S3. Aqua regia extractable phosphorus. Microwave-assisted aqua regia digestible phosphorus concentrations are reported for the IKMHSS tailings samples with respect to treatment and time.

Treatment	Depth[b] (cm)	P Concentration (mmol kg^{-1})[a]			
		2010	2011	2012	2013
0%	0–20	18 (1)	18 (1)	18 (4)	22 (4)
0%	20–40	33 (5)	--[c]	29 (6)	40 (8)
0%	40–60	28 (3)	26 (1)	28 (5)	30 (3)
0%	60–90	30 (2)	24 (1)	26 (0)	31 (2)
10% & Seed	0–20	41 (7)	47 (10)	49 (17)	53 (7)
10% & Seed	20–40	32 (6)	31 (4)	25 (2)	33 (5)
10% & Seed	40–60	30 (4)	24 (3)	22 (2)	26 (2)
10% & Seed	60–90	32 (4)	23 (3)	25 (3)	2 (6)
20%	0–20	74 (19)	54 (33)	44 (7)	66 (16)
20%	20–40	30 (3)	31 (4)	36 (11)	60 (56)
20%	40–60	29 (2)	24 (2)	24 (3)	36 (17)
20%	60–90	34 (2)	23 (2)	20 (1)	32 (3)
20% & Seed	0–20	42 (27)	52 (6)	52 (10)	64 (16)
20% & Seed	20–40	32 (4)	32 (4)	30 (4)	34 (8)
20% & Seed	40–60	28 (3)	23 (3)	24 (2)	28 (2)
20% & Seed	60–90	33 (4)	23 (1)	22 (1)	29 (3)

[a] Extraction performed according to EPA method 3051A. Standard deviation for n = 4 field replicates reported in parentheses.
[b] Depth is the composited interval below the surface.
[c] Measurements not collected.

Table S4. Aqua regia extractable vanadium. Microwave-assisted aqua regia digestible vanadium concentrations are reported for the IKMHSS tailings samples with respect to treatment and time. Values were used as the elemental total in sequential extraction calculations (Figure 1, Figure S4).

Treatment	Depth[b] (cm)	V Concentration (mmol kg^{-1})[a]			
		2010	2011	2012	2013
0%	0–20	0.91 (0.09)	0.81 (0.26)	0.75 (0.09)	1.41 (0.24)
0%	20–40	0.82 (0.13)	0.88 (0.15)	1.24 (0.23)	0.83 (0.08)
0%	40–60	0.84 (0.10)	1.07 (0.09)	1.14 (0.08)	0.90 (0.17)
0%	60–90	2.21 (0.27)	0.89 (0.07)	1.45 (0.20)	2.07 (0.35)
10% & Seed	0–20	1.09 (0.14)	1.10 (0.20)	1.08 (0.15)	1.42 (0.18)
10% & Seed	20–40	0.77 (0.06)	0.83 (0.12)	1.04 (0.08)	0.76 (0.07)
10% & Seed	40–60	0.85 (0.22)	0.97 (0.12)	1.22 (0.18)	0.85 (0.17)
10% & Seed	60–90	2.10 (0.39)	0.99 (0.11)	1.43 (0.40)	2.17 (0.43)
20%	0–20	0.92 (0.08)	0.86 (0.17)	0.76 (0.04)	1.53 (0.18)
20%	20–40	0.92 (0.12)	1.11 (0.16)	1.24 (0.17)	0.82 (0.19)
20%	40–60	1.14 (0.22)	1.15 (0.13)	1.40 (0.11)	1.36 (0.15)
20%	60–90	2.11 (0.28)	1.12 (0.21)	1.09 (0.12)	2.09 (0.41)
20% & Seed	0–20	0.91 (0.14)	0.91 (0.15)	0.80 (0.14)	1.42 (0.28)
20% & Seed	20–40	0.80 (0.10)	1.19 (0.16)	1.23 (0.22)	0.81 (0.08)
20% & Seed	40–60	1.06 (0.04)	1.30 (0.36)	1.26 (0.29)	1.24 (0.18)
20% & Seed	60–90	2.26 (0.38)	1.24 (0.11)	1.31 (0.02)	1.92 (0.26)

[a] Extraction performed according to EPA method 3051A. Standard deviation for n = 4 field replicates reported in parentheses.
[b] Depth is the composited interval below the surface.

Table S5. Aqua regia extractable chromium. Microwave-assisted aqua regia digestible chromium concentrations are reported for the IKMHSS tailings samples with respect to treatment and time. Values were used as the elemental total in sequential extraction calculations (Figure 1, Figure S5).

Treatment	Depth[b] (cm)	Cr Concentration (mmol kg^{-1})[a]			
		2010	2011	2012	2013
0%	0–20	0.40 (0.08)	0.32 (0.04)	0.28 (0.06)	0.38 (0.09)
0%	20–40	0.29 (0.20)	0.39 (0.19)	0.51 (0.21)	0.31 (0.18)
0%	40–60	0.41 (0.04)	0.43 (0.14)	0.42 (0.14)	0.40 (0.15)
0%	60–90	0.56 (0.07)	0.37 (0.04)	0.52 (0.11)	0.53 (0.12)
10% & Seed	0–20	0.48 (0.09)	0.41 (0.05)	0.39 (0.03)	0.44 (0.05)
10% & Seed	20–40	0.26 (0.05)	0.46 (0.06)	0.40 (0.09)	0.27 (0.09)
10% & Seed	40–60	0.41 (0.11)	0.53 (0.05)	0.49 (0.08)	0.42 (0.07)
10% & Seed	60–90	0.63 (0.14)	0.53 (0.06)	0.65 (0.21)	0.67 (0.16)
20%	0–20	0.34 (0.05)	0.35 (0.02)	0.31 (0.05)	0.39 (0.06)
20%	20–40	0.76 (0.07)	0.54 (0.16)	0.55 (0.28)	0.64 (0.08)
20%	40–60	0.52 (0.09)	0.55 (0.04)	0.57 (0.04)	0.60 (0.05)
20%	60–90	0.64 (0.12)	0.52 (0.11)	0.43 (0.04)	0.67 (0.18)
20% & Seed	0–20	0.39 (0.08)	0.40 (0.12)	0.37 (0.10)	0.42 (0.10)
20% & Seed	20–40	0.07 (0.13)	0.47 (0.12)	0.44 (0.05)	0.15 (0.29)
20% & Seed	40–60	0.40 (0.05)	0.50 (0.17)	0.48 (0.14)	0.48 (0.15)
20% & Seed	60–90	0.70 (0.11)	0.52 (0.06)	0.55 (0.03)	0.61 (0.04)

[a] Extraction performed according to EPA method 3051A. Standard deviation for n = 4 field replicates reported in parentheses.
[b] Depth is the composited interval below the surface.

Table S6. Aqua regia extractable manganese. Microwave-assisted aqua regia digestible manganese concentrations are reported for the IKMHSS tailings samples with respect to treatment and time. Values were used as the elemental total in sequential extraction calculations (Figure 1, Figure S6).

Treatment	Depth (cm)[b]	Mn Concentration (mmol kg^{-1})[a]			
		2010	2011	2012	2013
0%	0–20	6.4 (3.0)	3.9 (0.8)	3.5 (0.4)	3.4 (0.6)
0%	20–40	23 (6)	14 (7)	19 (11)	16 (8)
0%	40–60	30 (13)	31 (7)	24 (7)	32 (14)
0%	60–90	16 (1)	19 (2)	30 (7)	25 (9)
10% & Seed	0–20	10.4 (4.7)	7.1 (5.9)	4.2 (0.8)	3.8 (0.5)
10% & Seed	20–40	25 (9)	14 (13)	12 (10)	14 (12)
10% & Seed	40–60	24 (3)	27 (7)	23 (8)	30 (11)
10% & Seed	60–90	15 (2)	19 (3)	26 (9)	23 (9)
20%	0–20	6.5 (1.4)	7.5 (2.6)	5.2 (1.3)	4.8 (0.8)
20%	20–40	23 (5)	19 (4)	23 (13)	12 (6)
20%	40–60	32 (14)	30 (13)	32 (12)	39 (17)
20%	60–90	18 (4)	16 (3)	22 (3)	18 (6)
20% & Seed	0–20	7.8 (2.9)	5.3 (1.1)	6.2 (2.0)	4.7 (1.6)
20% & Seed	20–40	26 (7)	18 (4)	18 (11)	8.3 (4.5)
20% & Seed	40–60	23 (3)	30 (6)	23 (9)	35 (13)
20% & Seed	60–90	17 (1)	22 (4)	17 (7)	20 (5)

[a] Extraction performed according to EPA method 3051A. Standard deviation for n = 4 field replicates reported in parentheses.
[b] Depth is the composited interval below the surface.

Table S7. Aqua regia extractable iron. Microwave-assisted aqua regia digestible iron concentrations are reported for the IKMHSS tailings samples with respect to treatment and time. Values were used as the elemental total in sequential extraction calculations (Figure 1, Figure S7).

Treatment	Depth[b] (cm)	Fe Concentration (mmol kg^{-1})[a]			
		2010	2011	2012	2013
0%	0–20	1900 (200)	1600 (200)	1700 (200)	1800 (70)
0%	20–40	2300 (200)	2200 (200)	2000 (200)	2400 (100)
0%	40–60	2600 (500)	2200 (100)	2200 (200)	2500 (100)
0%	60–90	2500 (100)	2200 (100)	2300 (200)	2400 (200)
10% & Seed	0–20	2100 (200)	1800 (400)	1800 (100)	1800 (300)
10% & Seed	20–40	2300 (100)	2000 (200)	2000 (200)	2300 (200)
10% & Seed	40–60	2200 (400)	2100 (60)	2300 (100)	2400 (300)
10% & Seed	60–90	2500 (100)	2200 (200)	2200 (200)	2500 (100)
20%	0–20	1800 (70)	1700 (300)	1600 (200)	1800 (100)
20%	20–40	2100 (200)	2100 (100)	2200 (200)	2000 (100)
20%	40–60	2500 (300)	2100 (200)	2100 (200)	2200 (200)
20%	60–90	2800 (400)	2300 (70)	2400 (200)	2600 (100)
20% & Seed	0–20	2100 (300)	1900 (100)	1700 (200)	1800 (100)
20% & Seed	20–40	2100(100)	2100 (100)	2200 (100)	1900 (200)
20% & Seed	40–60	2500 (300)	2100 (400)	2300 (300)	2200 (100)
20% & Seed	60–90	2800 (500)	2300 (50)	2200 (300)	2600 (200)

[a] Extraction performed according to EPA method 3051A. Standard deviation for n = 4 field replicates reported in parentheses.
[b] Depth is the composited interval below the surface.

Table S8. Aqua regia extractable cobalt. Microwave-assisted aqua regia digestible cobalt concentrations are reported for the IKMHSS tailings samples with respect to treatment and time. Values were used as the elemental total in sequential extraction calculations (Figure 1, Figure S8).

Treatment	Depth[b] (cm)	Co Concentration (mmol kg^{-1})[a]			
		2010	2011	2012	2013
0%	0–20	0.16 (0.05)	0.09 (0.01)	0.08 (0.02)	0.12 (0.03)
0%	20–40	0.43 (0.09)	0.31 (0.06)	0.38 (0.15)	0.32 (0.11)
0%	40–60	0.60 (0.13)	0.53 (0.10)	0.50 (0.06)	0.59 (0.18)
0%	60–90	0.46 (0.07)	0.41 (0.03)	0.55 (0.16)	0.65 (0.16)
10% & Seed	0–20	0.24 (0.10)	0.16 (0.13)	0.11 (0.01)	0.13 (0.03)
10% & Seed	20–40	0.44 (0.11)	0.31 (0.17)	0.30 (0.13)	0.29 (0.14)
10% & Seed	40–60	0.50 (0.14)	0.52 (0.13)	0.51 (0.04)	0.60 (0.10)
10% & Seed	60–90	0.50 (0.05)	0.47 (0.03)	0.56 (0.15)	0.67 (0.22)
20%	0–20	0.10 (0.02)	0.13 (0.06)	0.10 (0.03)	0.13 (0.02)
20%	20–40	0.42 (0.05)	0.40 (0.06)	0.35 (0.08)	0.23 (0.10)
20%	40–60	0.59 (0.19)	0.58 (0.21)	0.67 (0.13)	0.63 (0.20)
20%	60–90	0.55 (0.05)	0.42 (0.04)	0.46 (0.05)	0.58 (0.09)
20% & Seed	0–20	0.18 (0.06)	0.12 (0.04)	0.14 (0.05)	0.15 (0.03)
20% & Seed	20–40	0.43 (0.14)	0.35 (0.07)	0.40 (0.15)	0.20 (0.07)
20% & Seed	40–60	0.46 (0.04)	0.53 (0.06)	0.50 (0.10)	0.55 (0.14)
20% & Seed	60–90	0.56 (0.07)	0.46 (0.06)	0.46 (0.02)	0.57 (0.08)

[a] Extraction performed according to EPA method 3051A. Standard deviation for n = 4 field replicates reported in parentheses.
[b] Depth is the composited interval below the surface.

Table S9. Aqua regia extractable nickel. Microwave-assisted aqua regia digestible nickel concentrations are reported for the IKMHSS tailings samples with respect to treatment and time. Values were used as the elemental total in sequential extraction calculations (Figure 1, Figure S9).

		Ni Concentration (mmol kg^{-1})[a]			
**Treatment**	**Depth[b] (cm)**	**2010**	**2011**	**2012**	**2013**
0%	0–20	0.36 (0.06)	0.22 (0.10)	0.13 (0.01)	0.12 (0.03)
0%	20–40	0.28 (0.11)	0.25 (0.05)	0.30 (0.13)	0.22 (0.13)
0%	40–60	0.42 (0.15)	0.37 (0.11)	0.31 (0.02)	0.34 (0.15)
0%	60–90	0.31 (0.03)	0.30 (0.03)	0.41 (0.11)	0.38 (0.14)
10% & Seed	0–20	0.24 (0.07)	0.18 (0.06)	0.14 (0.03)	0.14 (0.01)
10% & Seed	20–40	0.29 (0.06)	0.33 (0.13)	0.23 (0.04)	0.23 (0.04)
10% & Seed	40–60	0.38 (0.09)	0.39 (0.05)	0.39 (0.07)	0.42 (0.11)
10% & Seed	60–90	0.31 (0.04)	0.36 (0.05)	0.45 (0.16)	0.39 (0.11)
20%	0–20	0.14 (0.01)	0.37 (0.12)	0.15 (0.02)	0.13 (0.02)
20%	20–40	0.30 (0.03)	0.32 (0.08)	0.29 (0.04)	0.21 (0.04)
20%	40–60	0.48 (0.17)	0.42 (0.13)	0.47 (0.08)	0.48 (0.14)
20%	60–90	0.33 (0.06)	0.34 (0.09)	0.34 (0.06)	0.37 (0.13)
20% & Seed	0–20	0.40 (0.04)	0.28 (0.11)	0.18 (0.06)	0.15 (0.03)
20% & Seed	20–40	0.27 (0.13)	0.27 (0.08)	0.30 (0.09)	0.18 (0.06)
20% & Seed	40–60	0.34 (0.07)	0.39 (0.13)	0.36 (0.13)	0.40 (0.14)
20% & Seed	60–90	0.38 (0.10)	0.35 (0.04)	0.37 (0.03)	0.37 (0.09)

[a] Extraction performed according to EPA method 3051A. Standard deviation for n = 4 field replicates reported in parentheses.
[b] Depth is the composited interval below the surface.

Table S10. Aqua regia extractable copper. Microwave-assisted aqua regia digestible copper concentrations are reported for the IKMHSS tailings samples with respect to treatment and time. Values were used as the elemental total in sequential extraction calculations (Figure 1, Figure S10).

		Cu Concentration (mmol kg^{-1})[a]			
**Treatment**	**Depth[b] (cm)**	**2010**	**2011**	**2012**	**2013**
0%	0–20	3.2 (0.6)	1.6 (0.2)	1.6 (0.3)	1.9 (0.8)
0%	20–40	3.7 (1.2)	3.0 (0.4)	3.7 (1.9)	3.5 (1.0)
0%	40–60	3.3 (0.4)	3.4 (0.8)	4.1 (0.9)	4.2 (1.2)
0%	60–90	3.3 (0.2)	3.3 (0.2)	3.4 (0.1)	3.1 (0.2)
10% & Seed	0–20	3.4 (1.6)	2.3 (1.1)	1.9 (0.2)	1.8 (0.3)
10% & Seed	20–40	3.1 (0.4)	3.2 (0.3)	3.4 (1.3)	4.4 (1.6)
10% & Seed	40–60	3.2 (0.3)	3.3 (0.1)	3.6 (0.5)	3.4 (0.4)
10% & Seed	60–90	3.0 (0.3)	3.2 (0.3)	3.2 (0.3)	3.0 (0.4)
20%	0–20	1.8 (0.5)	2.4 (0.6)	2.0 (0.6)	1.8 (0.4)
20%	20–40	3.5 (0.6)	3.2 (0.6)	4.1 (0.7)	3.7 (0.9)
20%	40–60	2.9 (0.2)	3.0 (0.2)	3.1 (0.4)	3.1 (0.2)
20%	60–90	3.4 (0.9)	3.2 (0.4)	3.4 (0.7)	3.1 (0.4
20% & Seed	0–20	3.8 (1.6)	2.4 (0.3)	2.6 (0.7)	2.2 (0.4)
20% & Seed	20–40	3.1 (0.5)	3.0 (0.2)	3.6 (1.2)	4.0 (2.5)
20% & Seed	40–60	3.6 (1.0)	3.6 (1.2)	3.6 (1.2)	3.8 (1.5)
20% & Seed	60–90	3.4 (0.5)	3.3 (0.4)	3.1 (0.1)	3.0 (0.1)

[a] Extraction performed according to EPA method 3051A. Standard deviation for n = 4 field replicates reported in parentheses.
[b] Depth is the composited interval below the surface.

Table S11. Aqua regia extractable zinc. Microwave-assisted aqua regia digestible zinc concentrations are reported for the IKMHSS tailings samples with respect to treatment and time. Values were used as the elemental total in sequential extraction calculations (Figure 1, Figure S11).

Treatment	Depth (cm)[b]	Zn Concentration (mmol kg^{-1})[a]			
		2010	2011	2012	2013
0%	0–20	56 (20)	27 (6)	28 (6)	38 (20)
0%	20–40	210 (60)	170 (80)	150 (80)	160 (90)
0%	40–60	200 (70)	120 (20)	200 (60)	210 (50)
0%	60–90	120 (20)	170 (40)	140 (50)	160 (40)
10% & Seed	0–20	96 (40)	59 (60)	36 (2)	32 (7)
10% & Seed	20–40	160 (40)	110 (60)	110 (60)	140 (100)
10% & Seed	40–60	140 (20)	180 (60)	180 (60)	200 (20)
10% & Seed	60–90	90 (30)	130 (20)	100 (30)	120 (50)
20%	0–20	51 (20)	60 (40)	40 (20)	41 (20)
20%	20–40	160 (30)	170 (40)	220 (70)	110 (70)
20%	40–60	140 (30)	170 (60)	210 (30)	180 (60)
20%	60–90	150 (60)	130 (30)	190 (10)	150 (60)
20% & Seed	0–20	84 (30)	39 (10)	44 (20)	42 (10)
20% & Seed	20–40	180 (30)	140 (30)	140 (40)	82 (30)
20% & Seed	40–60	200 (80)	200 (130)	190 (80)	180 (40)
20% & Seed	60–90	150 (20)	160 (60)	150 (40)	160 (20)

[a] Extraction performed according to EPA method 3051A. Standard deviation for n = 4 field replicates reported in parentheses.
[b] Depth is the composited interval below the surface.

Table S12. Aqua regia extractable lead. Microwave-assisted aqua regia digestible lead concentrations are reported for the IKMHSS tailings samples with respect to treatment and time. Values were used as the elemental total in sequential extraction calculations (Figure 1, Figure S13).

Treatment	Depth (cm)[b]	Pb Concentration (mmol kg^{-1})[a]			
		2010	2011	2012	2013
0%	0–20	13 (2)	12 (3)	14 (3)	12 (2)
0%	20–40	13 (2)	12 (2)	12 (1)	13 (1)
0%	40–60	13 (2)	11 (2)	14 (2)	13 (1)
0%	60–90	12 (1)	13 (1)	12 (1)	11 (2)
10% & Seed	0–20	11 (1)	11 (1)	11 (1)	9.6 (0.3)
10% & Seed	20–40	13 (1)	10 (1)	11 (2)	12 (1)
10% & Seed	40–60	13 (1)	11 (2)	12 (1)	12 (1)
10% & Seed	60–90	11 (1)	12 (1)	11 (2)	11 (1)
20%	0–20	9.4 (1.6)	11 (3)	10 (1)	9.0 (1.7)
20%	20–40	13 (2)	11 (2)	11 (3)	11 (1)
20%	40–60	11 (0)	11 (1)	11 (2)	11 (0)
20%	60–90	13 (3)	12 (2)	12 (1)	12 (2)
20% & Seed	0–20	13 (3)	11 (1)	11 (1)	9.6 (0.7)
20% & Seed	20–40	13 (0)	11 (1)	11 (1)	11 (2)
20% & Seed	40–60	14 (3)	12 (4)	12 (3)	12 (1)
20% & Seed	60–90	12 (3)	12 (1)	11 (0)	12 (1)

[a] Extraction performed according to EPA method 3051A. Standard deviation for n = 4 field replicates reported in parentheses.
[b] Depth is the composited interval below the surface.
